# Proving the automatic benchtop electrochemical station for the development of dopamine and paracetamol sensors

**DOI:** 10.1007/s00604-024-06454-6

**Published:** 2024-06-20

**Authors:** Marek Haššo, Jiří Kudr, Jan Zítka, Jan Šílený, Pavel Švec, Ľubomír Švorc, Ondřej Zítka

**Affiliations:** 1 Institute of Analytical Chemistry, Faculty of Chemical and Food Technology, Slovak University of Technology in Bratislava, Radlinského 9, Bratislava, 812 37 Slovakia; 2 Department of Chemistry and Biochemistry, Mendel University in Brno, Zemedelska 1, Brno, 613 00 Czech Republic

**Keywords:** Acetaminophen, Electrodeposition, Screen-printed electrode, Differential pulse voltammetry, Electroplating, Sensor array

## Abstract

The introduced work represents an implementation of the automatic benchtop electrochemical station (BES) as an effective tool for the possibilities of high-throughput preparation of modified sensor/biosensors, speeding up the development of the analytical method, and automation of the analytical procedure for the determination of paracetamol (PAR) and dopamine (DOP) as target analytes. Within the preparation of gold nanoparticles modified screen-printed carbon electrode (AuNPs-SPCE) by electrodeposition, the deposition potential *E*_DEP_, the deposition time *t*_DEP_, and the concentration of HAuCl_4_ were optimized and their influence was monitored on 1 mM [Ru(NH_3_)_6_]^3+/2+^ redox probe and 50 μM DOP. The morphology of the AuNPs-SPCE prepared at various modification conditions was observed by SEM. The analytical performance of the AuNPs-SPCE prepared at different modification conditions was evaluated by a construction of the calibration curves of DOP and PAR. SPCE and AuNPs-SPCE at modification condition providing the best sensitivity to PAR and DOP, were successfully used to determine PAR and DOP in tap water by “spike-recovery” approach. The BES yields better reproducibility of the preparation of AuNPs-SPCE (RSD = 3.0%) in comparison with the case when AuNPs-SPCE was prepared manually by highly skilled laboratory operator (RSD = 7.0%).

**Graphical abstract:**

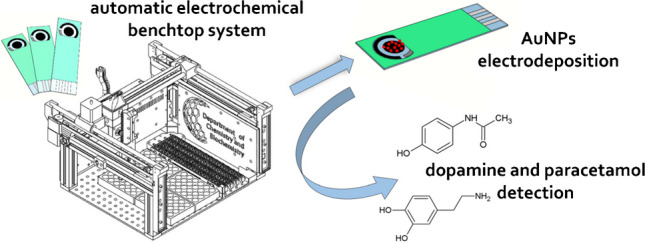

**Supplementary Information:**

The online version contains supplementary material available at 10.1007/s00604-024-06454-6.

## Introduction

Nowadays, nanomaterials have a broad range of applications including environmental and food safety, medical technologies, energy conversion and electronics, etc. They attract attention of researchers due to their fascinating physico-chemical properties. Among others, gold nanoparticles (AuNPs) represent a promising material for electroanalytical chemistry especially for biosensors development and sensors surface modification [1]. In this sense, they benefit from excellent conductivity, high surface to volume ratio, and favorable catalytic properties compared to bulk gold counterpart [2]. In addition, AuNPs roughen the electrodes surface and enhance mass transport [3]. AuNPs are also able to decrease of overpotentials of many redox reactions and maintain reaction reversibility [4]. These nanoparticles can be used to covalently immobilize organosulfur-containing recognition biomolecules such as oligonucleotides, aptamers, peptides, or antibodies, hence are often used in the field of biosensors to create self-assembled monolayers (SAMs) [5–7]. In case of biosensors, a high surface-to-volume ratio of AuNPs can increase recognition biomolecule loading, hence improving their analytical parameters [8, 9]. Various methods of AuNP-based electrode fabrication such as electrodeposition, electrostatic attachment during drop-casting or immersion into solution of alternatively fabricated NPs, and mixing AuNPs into paste of paste electrodes were previously reported [10–12]. Apart from mentioned, electrodeposition represents the straightforward, low-cost, and fast approach since no additional chemical synthesis equipment is necessary [13]. It represents the process of reduction of metal ions in electrolyte to elemental form (except metal oxides), which results in metallic, bimetallic, or trimetallic alloyed structures creation on particular working electrode surface [14–16]. Indubitably, electrochemical approaches for direct fabrication of nanomaterials on electrodes are first choices for electrochemists since they provide them extraordinary control over amounts, sizes, and shapes of deposited nanoparticles by well-known methods [17]. Regarding shapes of electrodeposited nanomaterials, spheres, cubes, stars, flowers, rods, etc. were previously reported [18]. Electrodeposition excels in deposits homogeneity and provides nanomaterials rigidly attached to electrodes surfaces, which can be hardly achieved by nanomaterials fabricated by another approach. Further, chemically fabricated nanomaterials are often covered with capping agents, which make them stable against aggregation; however, they can negatively affect desired surface chemistry of nanomaterials. On the other hand, no capping agents are commonly used during electrodeposition [19]. Nanostructured gold deposits can also create microelectrode arrays by electrodeposition as was reported by Podešva et al. [20].

Credibility and integrity of scientific research are serious topic, where reproducibility of experimental data plays a substantial role. To obtain the set of scientifically sound experimental data is often expensive, time-consuming, and demanding, which can result in limited number of data points, insufficient repetitions, and ambiguous conclusions. Automation focuses on replacing of manual error-prone processes and provides more accurate, precise and consistent results [21]. Further, higher number of experimental data can lead to more comprehensive and solid conclusions. Nowadays, laboratory automation represents a complex integration of robotics, computing, liquid handling, and other technologies which focus on saving time and improving performance and thus statistical proof. Originally, laboratory automation has started to develop due to demands of clinical laboratories, for fast analysis of myriad of samples [22]. Second relevant field is pharmacy, where large number of samples are tested in order to find new biologically active compounds (high-throughput screening) [22]. Worth noting, a degree of automation in analytical and bioanalytical laboratories are far behind clinical laboratories and pharmaceutical industry. The reason is that analytical processes are highly variable and often comprise sample pretreatment to deal with matrix effect of complex samples [21]. Regarding the electrochemistry, high-throughput experimentation (HTE) is booming due to resurgence of electrosynthesis as a modern tool of organic synthesis [23]. Beside organic synthesis, high-throughput electrochemistry concept is mainly adopted in the field of battery and fuel cell research [24, 25]. The change for electroanalytical chemistry brought development of screen-printed electrodes (SPEs) and especially multielectrodes (electrode arrays) [26, 27]. Screen-printed sensors represent a cheap alternative to macroscopic electrodes, where restoration of electrode active surface is omitted by its disposability. Current trends in the field of SPE biosensors belong modification of their surface with imprinted polymers (synthetic recognition elements), either chemically or electrochemically polymerized [28, 29]. Screen-printed multielectrode arrays either in two electrode or three electrode setups have been used as PCR products sensor, impedance gas sensor, in purpose of enzyme libraries screening and as immunosensor [30–33]. One approach to electrochemistry automation is based on creation of (micro)fluidic devices which includes many integral parts such as pumps, valves, and degassers [34, 35]. Here, automation is more focused on sample handling than on repetitive electrodes modification and analysis execution. Our unique approach to high-throughput electrochemistry is based on unique BES and related software that enable facile and reliable electrochemical analysis and individual SPEs processing in fully automatic mode. This approach is in general fully described at [36] and belongs to our continuous work in this field of electrochemical analysis automation [37, 38].

Herein, we present the detailed study of automatic AuNP electrodeposition on commercially obtained SPEs and their performance towards two model analytes with well-known redox behavior—DOP and PAR using BES.

## Experimental

### Materials

DOP (purity > 97.5%), PAR (98%), [Ru(NH_3_)_6_]Cl_3_ (98%), and KCl (99%) were of analytical purity and were bought from Sigma-Aldrich (St. Louis, MO, USA). HAuCl_4_ · xH_2_O (~ 50% Au basis) was bought from Sigma-Aldrich (St. Louis, MO, USA). The aqueous solutions were prepared in deionized water with the resistivity of higher than 18.2 MΩ·cm at 25 °C from Millipore (Burlington, MA, USA). Britton-Robinson (BR) buffer, composed of a mixture of H_3_BO_3_ (Lach-Ner, Neratovice, Czech Republic, p.a.), CH_3_COOH (St. Louis, MO, USA, puriss), and H_3_PO_4_ (Lach-Ner, Neratovice, Czech Republic, 85%) (each compound in concentration of 0.04 M), was used as supporting electrolyte. Various pH values were adjusted by adding 0.2 M NaOH (Lach-Ner, Neratovice, Czech Republic, p.a.) until BR buffer with the appropriate pH value was prepared. The stock solution of HAuCl_4_ was prepared at higher concentration (app. 0.05 M) by dissolving certain amount of HAuCl_4_ · xH_2_O in 10 mL of deionized water, transferred to 25-mL volumetric flask, and filled up with deionized water. The working solution of 1 mM HAuCl_4_ for deposition step was prepared by a dilution of the suitable volume of HAuCl_4_ stock solution with 0.1 mM H_2_SO_4_ (St. Louis, MO, USA, 95–98%) in 10-mL volumetric flask. The 1 mM stock solutions of DOP and PAR, used for the preparation of working solutions at low concentration levels (micromolar), were prepared by dissolving suitable amount of reference material in small amount of deionized water and were transferred to 50-mL volumetric flask.

### Instruments

**Fig. 1:**
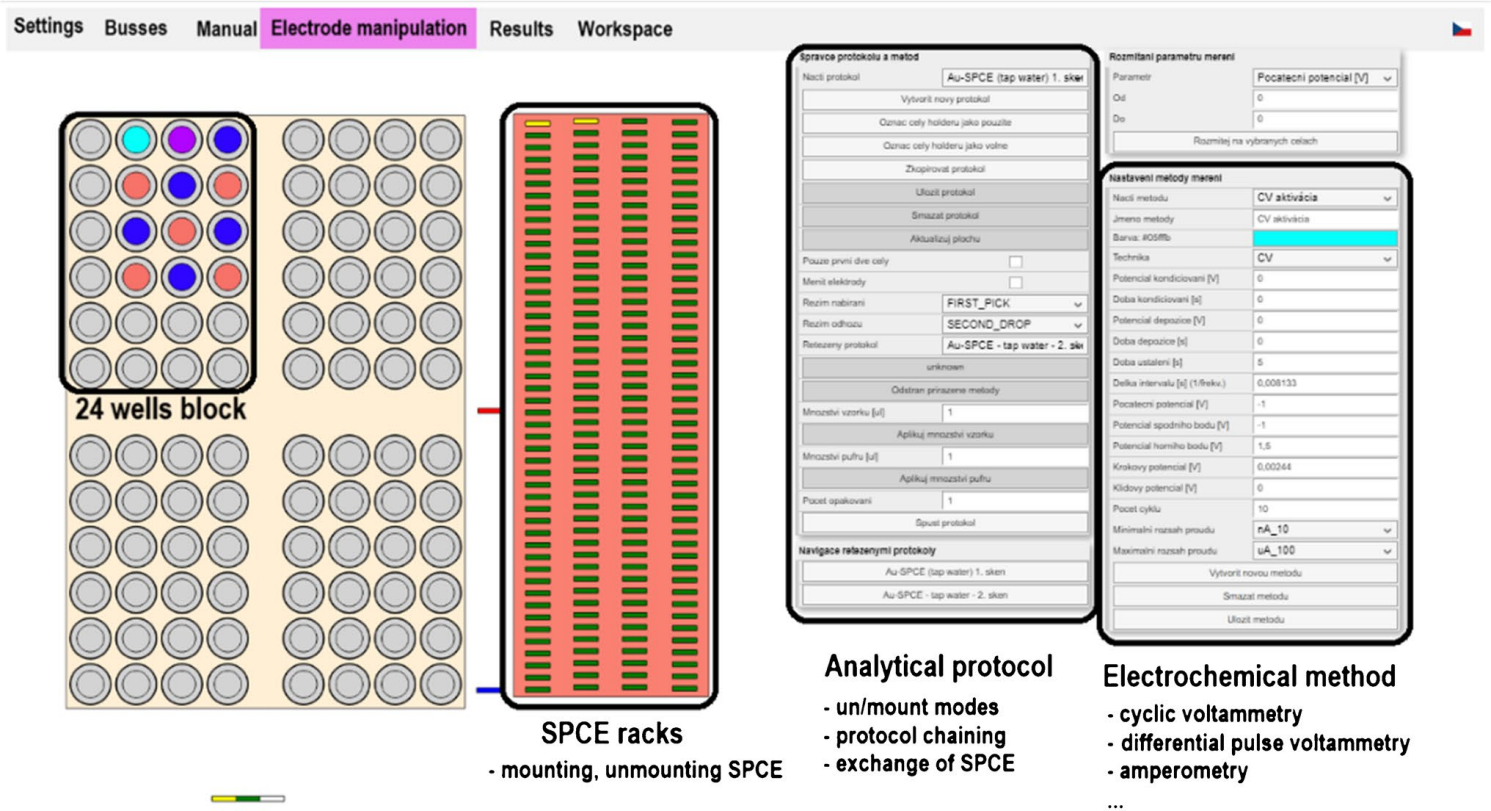
Image of the software used for the control of BES and creation of analytical protocols with electrochemical methods assigned to certain wells

All measurements were performed by the prototype of the automatic BES, which was designed to work in three various working modes (i) submerging mode, (ii) flow injection mode, and (iii) drop-casting mode. In this study, the BES was working in submerging mode. This station was accomplished in the system integrated minipotentiostat EmStat 4 (PalmsSens, Houten, The Netherlands) interfaced to station inherent software (Fig. 1) [36]. In the case of electrochemical measurements realized by laboratory operator, potentiostat PalmSens 4 (PalmSesns, Houten, The Netherlands) was used. The electrochemical measurements were performed using the screen-printed carbon electrodes (SPCE) obtained from Micrux technologies (Gijón, Spain). The body of SPCE electrode is made of PET substrate on which a carbon working electrode (diameter of 3 mm), silver pseudoreference electrode, and carbon auxiliary electrode are placed. During the electrochemical procedures, when the BES was working in submerging mode, the particular SPCEs were immersed in tissue culture plates with 24 wells at app. 5 mL volume of well from Jet Biofil (Guangzhou, China).

### Electrochemical measurements

Cyclic voltammetry (CV) was used to evaluate the influence of the modification conditions on the voltammetric responses of 1 mM [Ru(NH_3_)_6_]Cl_3_ in 0.1 M KCl and 50 μM DOP in BR pH 4.0. The study of the voltammetric behavior of DOP on the bare SPCE and the AuNPs-SPCE at various scan rates and the influence of pH on the voltammetric responses of DOP and PAR was also realized by CV. For the electrodeposition of AuNPs on the surface of the SPCE, amperometry at the fixed detection potential (deposition potential—*E*_DEP_) for a certain period of time (deposition time—*t*_DEP_) was used. Differential pulse voltammetry (DPV) was used as a detection technique to determine PAR in the real-sample analysis, for the evaluation of the influence of modification conditions on the analytical performance of AuNPs-SPCE and the pH study of DOP and PAR.

### SEM measurements

The morphology of electrodes was examined by scanning electron microscopy on a Tescan MAIA 3 equipped with a field emission gun (Tescan Ltd., Brno, Czech Republic). The most appropriate pictures were recorded using the In-Lens secondary electron (SE) and back-scattered electron (BSE) detectors at a working distance around 3.00 mm and at 5 kV acceleration voltage. 768 × 858 pixel images were obtained at 25,000–100,000-fold magnification covering a sample area of 2.08^2^–8.30^2^ μm^2^. Full frame capture was performed in UH Resolution mode. The accumulation of image with image shift correction was enabled and it took about 30 s with the ∼ 0.32 μs/pixel dwell time. The spot size was set to 2.5 nm.

The particle distribution analysis was performed using ImageJ software (www.imagej.net). Micrographs of suitable resolution were processed with the band-pass filter. Subsequently, the threshold of images was adjusted and the particle analysis with suitable upper and lower cutoff was performed. From obtained cross-sectional areas of all particles presented within the image, diameters of circularly shaped particles were expressed in histograms.

### Description of electrochemical station

The automatic electrochemical station is based on 3-axis position system. It is able to mount SPE electrodes in electrode rack, move it to predefined well filled with electrolyte or sample, perform their electrochemical method such as CV, DPV, and amperometry or just dip electrode into the well to wash it. Subsequently, the used electrodes can be unmounted and released to the release tray. The mentioned processes can be used multiple-times with one SPE and can be repeated within one procedure sequence; hence, operator possesses significant degree of freedom to modify electrodes and/or perform analysis. The electrodes are mounted and unmounted into special electrodes head equipped with 3-pin and 4-pin FCC connector, which means that various SPE designs are compatible with the station. The station contains two commercial potentiostats EmStat 4 (PalmsSens, Houten, The Netherlands). Electrode tray can be filled with 96 SPEs and working place of station can be filled with four 24-well titration plates. Further details are described in *Anal. Methods*, 2022, 14, 3824 [36]. All experiments, except for manual real sample analysis and manual modification, were performed using this automated benchtop system. This represents practical analytical application in the field, facilitating automated analysis, electrode modification, and expediting the method development process.

## Results and discussion

### Electrodeposition of gold nanoparticles on screen-printed carbon electrodes

The growth mechanism of gold nanoparticles by electrochemical reduction involves the reduction of Au(III) in tetrachloroauric acid to metallic gold atoms (Au(0)). This reduction process leads to the formation of stable gold clusters or nuclei, which are the initial building blocks for the growth of gold nanoparticles. The size, shape, and structure of the resulting gold nanoparticles are influenced by the electrochemical conditions, highlighting the importance of controlling these parameters of used electrochemical method for desired nanoparticle properties [39, 40]. Many published articles dealing with the modification of the working electrode by electrodeposition have utilized cyclic voltammetry as an electrochemical technique [41]. Regardless of the electrochemical technique used (cyclic voltammetry or amperometry), the same principle of the electrochemical reduction of Au(III) to Au(0), formation of gold nuclei, and subsequent growth to nanoparticles is involved. The problem of using cyclic voltammetry lies in the fact, that the size of NPs and density of NPs on the surface is simultaneously influenced by number of CV scans. Instead of change on only instrumental parameters in the case of CV (number of scan). Because of this, the amperometry has been chosen due to its ability to provide the change of more studied modification parameter (*E*_DEP_ or *t*_DEP_) which influenced the size and abundance of NPs resulting in the better control process of modification step.

**Fig. 2:**
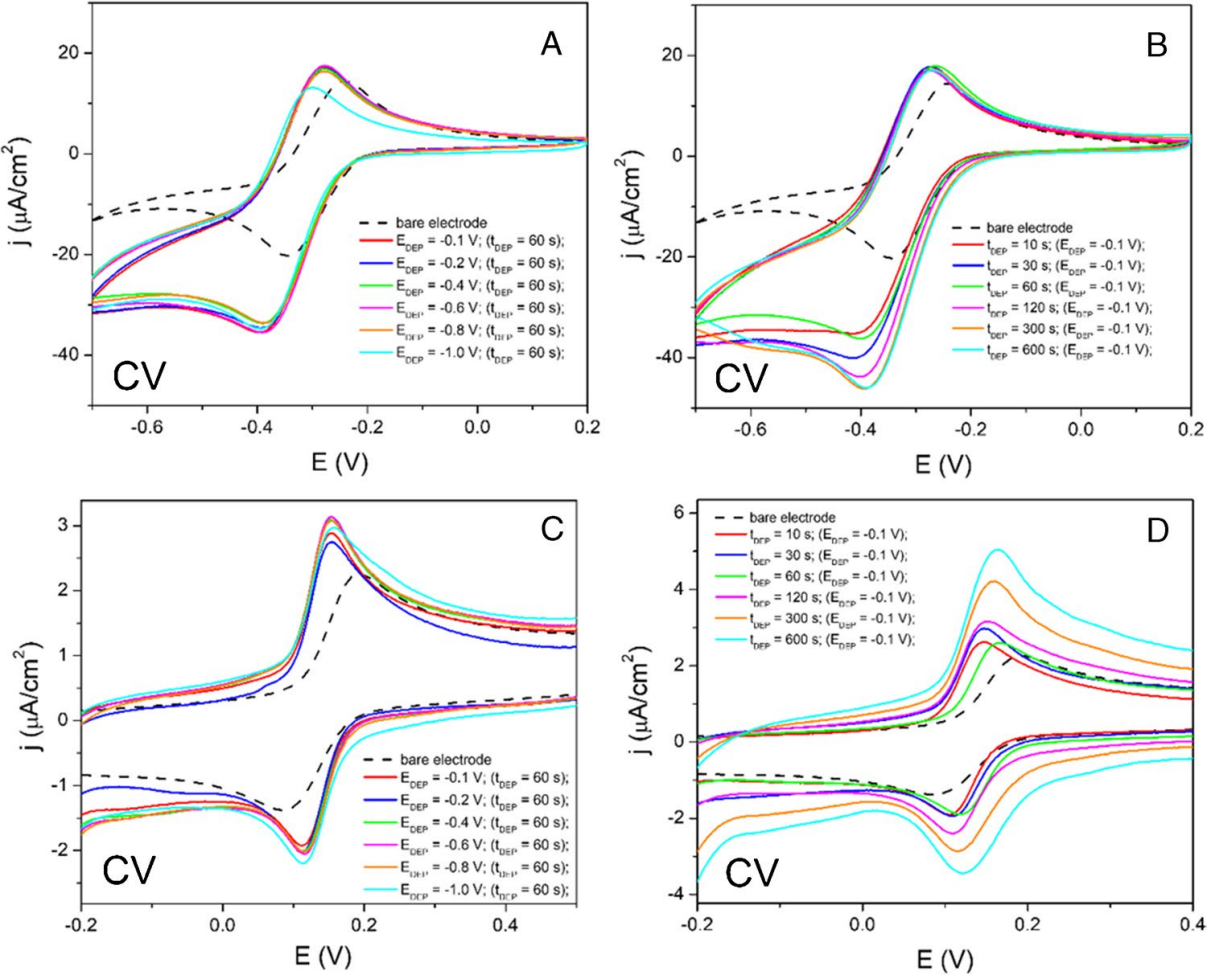
CV records of 1 mM [Ru(NH_3_)_6_]^3+/2+^ redox probe in 0.1 M KCl on SPCE and AuNPs-SPCE at various modifications conditions (*E*_DEP_, *t*_DEP_) with a scan rate of 100 mV/s (**A**, **B**). CV records of 50 μM DOP in BR pH 4.0 on SPCE and AuNPs-SPCE at various modifications conditions (*E*_DEP_, *t*_DEP_) (**C**, **D**) with a scan rate of 100 mV/s

In the initial step, the influence of several experimental conditions (deposition potential, deposition time, concentration of the deposited HAuCl_4_) on the AuNPs-SPCE prepared by electrodeposition was investigated, considering the AuNP morphology and the analytical performance of this sensor towards DOP as a model analyte. The effects of the mentioned modification conditions on the AuNPs-SPCE and the electrochemical properties were determined using [Ru(NH_3_)_6_]^3+/2+^ redox probe as well as the intensities of the analytical signal of DOP were evaluated. Figure 2 displays cyclic voltammograms of 1 mM [Ru(NH_3_)_6_]^3+/2+^ in 0.1 M KCl and 50 μM DOP in BR pH 4.0 recorded at the bare and the AuNPs-SPCE. As is evident from Fig. S1 (A, B, see Supplementary Material), that show corresponding current densities of oxidation/reduction peaks of 1 mM [Ru(NH_3_)_6_]^3+/2+^ redox probe, the influence of *E*_DEP_ did not provide an unequivocal trend in the term of the applied *E*_DEP_. The similar oxidation peak current densities were noticed across the *E*_DEP_ range from − 0.1 to − 0.6 V, with the decrease observed at the potentials below − 0.6 V. For the reduction peak of 1 mM [Ru(NH_3_)_6_]^3+/2+^, the comparable current densities were noticed at the whole range of *E*_DEP_. Additionally, the influence of *t*_DEP_ at the *E*_DEP_ of − 0.1 V offers clear trend in the case of oxidation and also reduction peak of 1 mM [Ru(NH_3_)_6_]^3+/2+^. Recorded current densities increased with the growing deposition time until *t*_DEP_ of 300 s, at which the registered current density was stabilized (reached plato).

The influence of concentration of HAuCl_4_ solution on the intensities of 1 mM [Ru(NH_3_)_6_]^3+/2+^ was observed in the concentration range from 0.1 to 5.0 mM. Interestingly, a comparable trend of the percentage increase of primary response of 1 mM [Ru(NH_3_)_6_]^3+/2+^ was noticed for the oxidation and reduction peak. Based on very similar percentage increasing of initial voltammetric responses in the concentration range from 2.5 to 5 mM (reaching the plato at higher concentration, see Fig. S2), 1 mM HAuCl_4_ was chosen as an optimal concentration of the deposition solution, because it renders the considerable increase of voltammetric response.

During the investigation of the influence of modification parameters on DOP as a model analyte, it was found that the oxidation peak grew with decreasing *E*_DEP_ until the value − 0.6 V and lower *E*_DEP_ values (− 0.8 V and − 1.0 V) led to diminished oxidation peak of DOP. The increasing *t*_DEP_ significantly enhanced the oxidation peak of DOP across all studied *t*_DEP_ values (Fig. S1 C, D), and at the *t*_DEP_ higher than 120 s resulted in the noticeable grow of the background current in cathodic scans, resulting in a diminished reduction peak of DOP. The wider range of the *t*_DEP_ had to be examined due to the growing oxidation peak observed from 10 to 600 s. However, at the *t*_DEP_ values higher than 1200 s, the oxidation peak of DOP did not increase so significantly as previously (Fig. S3 A, B), suggesting that the *t*_DEP_ values exceeding 1200 s were unnecessary for the improvement of sensitivity. When applying the *t*_DEP_ of 1200 s, another oxidation signal was noticed at + 0.075 V and became more prominent with the increased *t*_DEP_. For the reduction peak of DOP, the influence of *E*_DEP_ did not show a clear trend in terms of the intensity of the recorded analytical signal (Fig. S1 D). Based on the sufficient sensitivity and the possible problem with the presence of the oxidation peak at + 0.075 V vs Ag/AgCl), that can affect the shape of the recorded voltammetric response of DOP at higher concentration levels, the maximum *t*_DEP_ providing the most favorable voltammetric responses of DOP was 1200 s.

### Voltammetric behavior of dopamine on AuNPs-SPCE

After the optimization of deposition conditions in the process of preparation of AuNPs-SPCE, the detailed voltammetric study of dopamine as a model analyte was performed to assess the electroactive surface area of the bare or the modified electrode. As shown in Fig. S4, a remarkable influence of the modification of SPCE by AuNPs on the voltammetric response of DOP (modification conditions: *E*_DEP_ =  − 0.1 V; *t*_DEP_ = 60 s) was noticed, that was represented by improved slope value for anodic and cathodic process. According to the Randles–Sevcik equation, it is suggesting the increase of the electroactive surface area of AuNPs-SPCE (*E*_DEP_ =  − 0.1 V, *t*_DEP_ = 60 s) and we expect, that in longer deposition time (*t*_DEP_ > 60 s), the effect on increasing of electroactive surface area will be more prominent.

### Morphology of SPCE and AuNPs-SPCE

The surface morphology of the SPCE and the AuNPs-SPCEs prepared at different deposition conditions was characterized by SEM scanning in the SE and BES modes. Fig. S5 represents SEM image of the bare SPCE. While SE mode disclosed the surface topography of the working electrode on SPCE, BES mode provided information about the composition of the working electrode on SPCE. Elements with a high atomic number, in our case Au, yielded more backscatter electrons than elements with a low atomic number and that was why they appeared as bright points in the images. It is clear from Fig. S6, that in the case of AuNPs-SPCE electrodeposited at various *E*_DEP_ and fixed *t*_DEP_ = 60 s, the quantity of particles, their distribution, and average size fluctuated with lowering *E*_DEP_. As for particle size, with lower *E*_DEP_ average diameter of Au particles decreased, however on the other hand their quantity was increased (Fig. S7). In spite of the fact that the smaller Au particles were accomplished at lower *E*_DEP_ <  − 0.6 V, the worse size uniformity was noticed. In the case of the influence of *t*_DEP_ at the fixed *E*_DEP_ =  − 0.1 V on the morphology of AuNPs-SPCE, gradually increasing of *t*_DEP_ caused the better distribution of Au particles on the electrode surface. As is shown in Fig. S8 A, at *t*_DEP_ = 10 s, the electrode surface was covered by Au nanoparticles, indicating the fact that the generation of nanoparticles nuclei was starting instantaneously at the initial stage of particle formation. When a certain number of Au nuclei are formed, the following electrochemical reduction of AuCl_4_^−^ takes place preferentially on these formed nuclei and not on the bare electrode surface [42, 43]. The diameter of these Au particles raised with increasing of *t*_DEP_ and at higher *t*_DEP_ = 600 s this effect was the most prominent (Fig. S9). Furthermore, Au particles were not uniform and spherical, but of irregular shape. The increase of concentration of HAuCl_4_ in the deposited solution caused raising size of Au particles and influenced their overall shape (Fig. S10). At higher concentration c > 1.0 mM, Au particles exhibited “nanocluster” structure instead of spherical particles (Fig. S11). Therefore, it can be deducted that process of nucleation and growing of Au particles is highly controlled by concentration of deposition medium, deposition potential and deposition time and then resulting quantity and diameter of AuNPs can be adjusted by these deposition parameters.

### Electrochemical behavior of DOP and PAR on SPCE and AuNPs-SPCE

#### Electrochemical activity of DOP as a model analyte

The electrochemical activity of DOP was investigated in BR due to its broad pH range. It was verified that DOP underwent the electrochemical reaction on the SPCE within the pH range from 4.0 to 8.0, consistent with data documented in the scientific literature [44]. As is demonstrated in Fig. S12 (A, C), the voltammetric responses of 50 μM DOP on the bare SPCE slightly increased in the pH range from 4.0 to 6.0, reaching its maximum at pH 5.5 (2.35 μA/cm^2^). Furthermore, the peak potential (*E*_p_) of DOP was prominently shifted to the more negative potentials in the pH range from 4.0 to 5.0. Another slight shifts of the *E*_p_ of DOP to the more negative values of potentials in the pH range from 5.0 to 8.0 were also observed. According to the *E*_p_ = f(pH) dependence, within the pH range of 4 to 5, both SPCE and AuNPs-SPCE exhibited very similar slope values (− 0.115 V/pH (bare), − 0.130 V/pH (modified)). These values are approximately twice the theoretical value (− 0.059 V/pH), suggesting the exchange of 2 e^−^ and 4 H^+^ in this pH range. As seen in Fig. S12 (C, D), a noticeable change of the slope appeared at pH 5.0. The achieved slope for the SPCE and the AuNPs-SPCE in the pH range from 5.0 to 8.0, revealed that in the case of bare electrode 2 e^−^ and ½ H^+^ participated in electrochemical reaction. Compared with the AuNPs-SPCE (slope value of − 0.047 V/pH) the exchange of 2 e^−^ and 2 H^+^ can be expected. When the AuNPs-SPCE with modification condition (*E*_DEP_ =  − 0.1 V, *t*_DEP_ = 1200 s) was utilized to explore the electrochemical behavior of DOP, the highest electrochemical activity was noticed in BR with pH 4.0. In general, it can be stated that the electrochemical activity of DOP is more prefer and intensive in slightly acidic medium [45]. Based on our results, the BR with pH 4.0 was selected as a supporting electrolyte for other consecutive measurements.

### Electrochemical activity of PAR as a target analyte

**Fig. 3:**
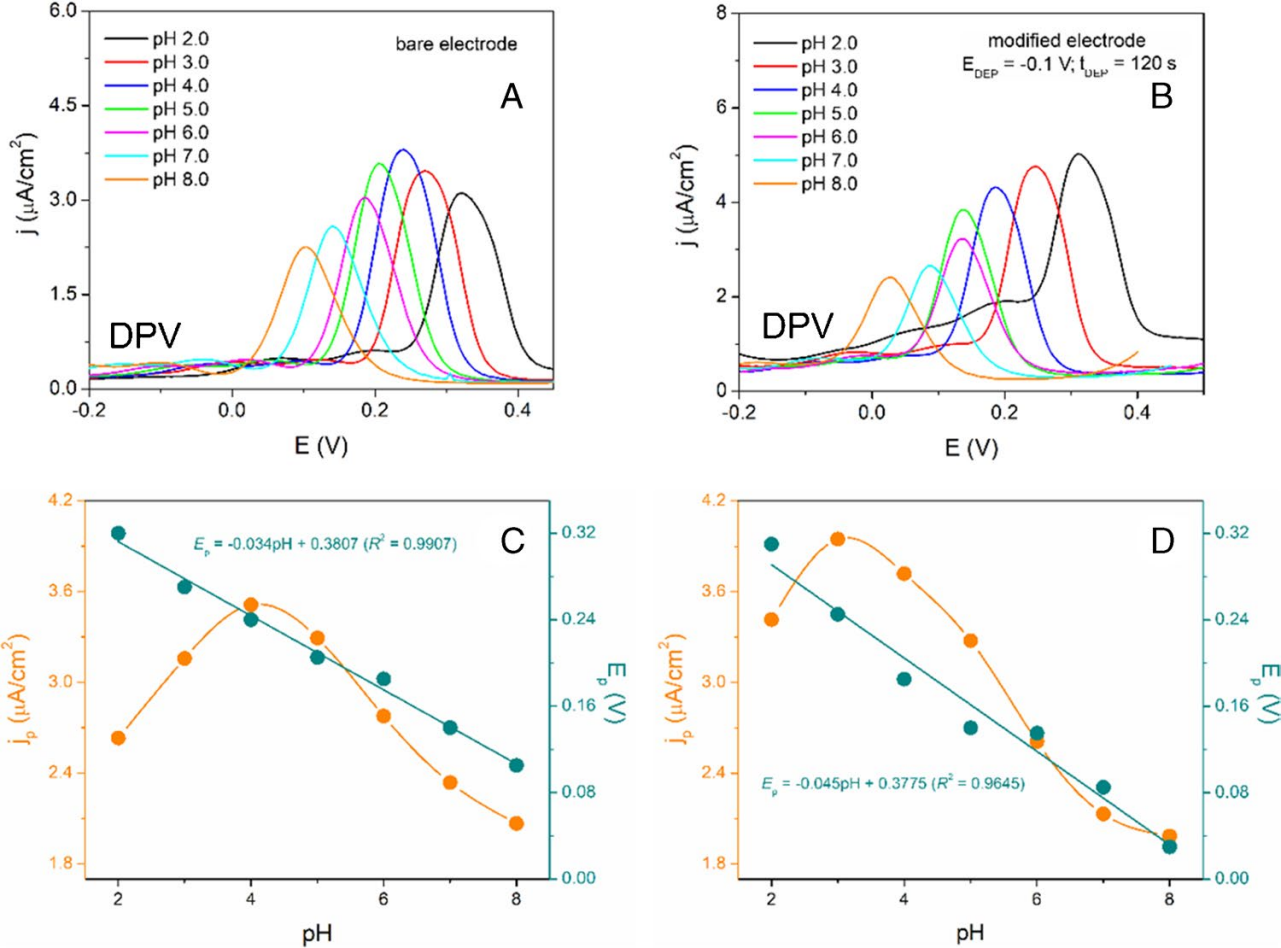
DP voltammograms of 50 μM PAR in BR buffer at different pH values (4.0–8.0) on bare SPCE (**A**) and modified SPCE (**B**). Dependence of current density (j_p_) and peak potential (*E*_p_) of 50 μM PAR on pH of BR buffer solution, recorded on bare SPCE (**C**) and modified SPCE (**D**). Pulse parameters: pulse height 100 mV, pulse time 100 ms, and interval time 0.5 s

The same as the electrochemical study of DOP mentioned in the previous section, the influence of supporting electrolyte pH on the electrochemical activity of paracetamol (PAR) was carried out in BR in the pH range from 2.0 to 8.0. It is clear from the DPV records in Fig. 3 that the AuNP-SPCE sensors showed higher voltammetric responses of PAR in more acidic medium (pH < 6.0). In addition, they exhibited a more prominent effect on the shifting of *E*_p_ of PAR when compared with the bare SPCE. This phenomenon indicates the electrochemical process of PAR in which 2 e^−^ and 2 H^+^ are exchanged, because the slope of *E*_p_ = f(pH) at – 0.045 V/pH was relatively close to the theoretical value of – 0.059 V/pH in the Nernst equation. On the other hand, in the case of the bare SPCE, the slope of dependence of *E*_p_ = f(pH) was found to be – 0.034 V/pH which is very close to the Nernstian value for 2 e^−^ and 1 H^+^ [46]. Taking into consideration the main purpose of this work and the applicability of AuNPs-SPCE in the process of developing of the analytical method, the BR pH 4.0 was chosen as a satisfactory supporting medium due to its favorable voltammetric response of PAR on the AuNPs-SPCE.

### Analytical performance of AuNP-SPCE sensor and its sensitivity in various modification conditions

**Fig. 4:**
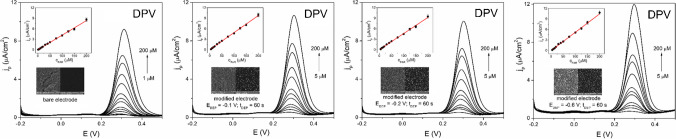
DP voltammograms of calibration solutions of PAR in the concentration ranges from 1 to 200 μM (bare SPCE) and 5 to 200 μM (modified electrodes at various conditions of *E*_DEP_) in BR pH 4.0 and their corresponding calibration curves (insets up), SEM images of bare SPCE and AuNPs-SPCE prepared at different *E*_DEP_ (insets down). Pulse parameters: pulse height 100 mV, pulse time 100 ms, and interval time 0.5 s

The influence of applied modification conditions on the performance of the proposed AuNP-SPCE sensor was evaluated by a construction of the calibration curves of DOP and PAR separately in various modifications conditions based on used *E*_DEP_ and *t*_DEP_. Subsequently, the essential analytical parameters were determined (Table 1 and Tables T1–T3, see Supplementary Material). When a *E*_DEP_ altered from − 0.1 V to − 1.0 V, the major influence was registered in the sensitivity (slope value of calibration curve) and narrower linear concentration range than in case of the bare SPCE (Fig. S13, Fig. S14). Concerning DOP as a model analyte, a sensitivity of the proposed sensors raised with lowering applied *E*_DEP_ until the value of − 0.6 V was set up since the comparable value of sensitivity were achieved in *E*_DEP_ lower than − 0.6 V. Based on this fact, it can be stated that lowering *E*_DEP_ did not prove the significant electrocatalytic effect on DOP, which is in good agreement with the results observed in section Electrodeposition of gold nanoparticles on screen-printed carbon electrodes. The influence of *t*_DEP_ on the analytical performance of the AuNPs-SPCE sensor for the determination of DOP revealed the increasing slope value of calibration curve until the *t*_DEP_ = 120 s and at *t*_DEP_ > 120 s a worse sensitivity of calibration curve was noticed (Fig. S15, Fig. S16). Similar to DOP, the sensitivity of the AuNP-SPCE sensors to PAR increased with lowering *E*_DEP_ to values of − 0.6 and at *E*_DEP_ ≤  − 0.6 V, sensitivities were comparable or even worse than those observed for DOP (Fig. 4, Fig. S17). On the other hand, the influence of *t*_DEP_ exhibited a clear trend, because the sensitivity of the prepared AuNP-SPCE sensor increased with the growing *t*_DEP_ (Fig. S18, Fig. S19). However, at *t*_DEP_ higher than 1200 s, the highest increase in the voltammetric response of PAR was noticed at the higher concentrations compared to the lower concentration levels of PAR and near the *E*_p_ of PAR, another tiny oxidation peak appeared at + 0.09 V. The provenance of this oxidation peak was not extensively studied. However, based on our observation, it can be assumed that this peak is related to the particular material of the SPCE used, as it was also detected in the supporting electrolyte and its intensity became more prominent with higher *t*_DEP_. In the case of DOP, the responses of the small signal at + 0.09 V were subtracted from the voltammetric responses of DOP at the different concentration levels, especially at low concentrations, in which its effect was more significant than at higher concentrations of DOP. Overall, the calibration curves provided good linearity with *R*^2^ > 0.991. However, regardless of the modification conditions, the AuNP-SPCE sensors yielded narrow linear concentration range for DOP and PAR. Another remarkable observation was that the lowest concentration levels of DOP (2.5 μM) and PAR (1 μM) were not detected at the AuNP-SPCE despite these sensors showing higher sensitivities in most cases. Some of obtained analytical parameters for AuNPs-SPCE prepared at *E*_DEP_ =  − 0.1 V and *t*_DEP_ = 120 s (DOP) and 1200 s (PAR) were compared with the newest analytical methods dealing with quantification of DOP and PAR on SPE modified by various modifiers (Table T4, see Supplementary Material) [47–58].

**Table 1:** The analytical parameters for the determination of PAR at the bare SPCE and the modified SPCE at various *E*_DEP_ (*n* = 3)

Parameter	Bare electrode	*E*_DEP_ = − 0.1 V	*E*_DEP_ = − 0.2 V	*E*_DEP_ = − 0.4 V	*E*_DEP_ = − 0.6 V	*E*_DEP_ = − 0.8 V	*E*_DEP_ = − 1.0 V
Intercept (μA/cm^2^)	0.048 ± 0.053	0.025 ± 0.061	0.037 ± 0.065	0.024 ± 0.072	− 0.052 ± 0.137	− 0.035 ± 0.121	− 0.128 ± 0.181
Slope (μA/cm^2^.μM)	0.042 ± 0.001	0.049 ± 0.001	0.046 ± 0.001	0.049 ± 0.001	0.053 ± 0.002	0.049 ± 0.001	0.053 ± 0.002
LCR (μM)	1–200	5–200	5–200	5–200	5–200	5–200	5–200
*R*^2^	0.9970	0.9979	0.9974	0.9971	0.9910	0.9913	0.993
LOD (μM)	3.8	3.8	4.2	4.5	7.8	7.4	10.3

### Application of SPCE and modified AuNP-SPCE sensors in the analysis of real water samples

The practical applicability of the SPCE and the modified AuNPs-SPCE manually and the BES was verified by the determination of PAR and DOP in real tap water sample by the standard addition method (Fig. S20 – S25). Since PAR was not detected in the particular sample and/or its eventual concentration in the sample was under the LOD, the spike-recovery assay was undertaken to evaluate the accuracy of the achieved results. The results are expressed as a confidence interval with 95% probability and are summarized in Table 2 and Table T5. In the manual approach, the determined amount of PAR in “spiked” tap water achieved by the bare SPCE and the AuNPs-SPCE were app. 92% and 96%, respectively. When the BES provided the modification step and following analysis, the determined amount of PAR in “spiked” tap water was 105% and 103%, respectively. The determined amount of DOP in “spiked” tap water yielded by the bare SPCE and the AuNPs-SPCE were app. 103.3% and 101%, respectively. According to these findings, it can be concluded that the presented bare SPCE and the AuNP-SPCE sensors are suitable to determine PAR and DOP in real water samples and the incorporation of the BES for the modification step of SPCE and followed analysis leads to the significant automation of analytical process and more accurate results.

**Table 2:** The determined amount of PAR in “spiked” tap water by the standard addition method for “spike-recovery” assay (*n* = 3)

Method of modification	Sensor	Sample/matrix	PAR added (μM)	PAR measured (μM)	Recovery (%)
-	SPCE	Tap water	30.0	27.7 ± 0.96	92.3
Manually	AuNPs-SPCE	Tap water	30.0	28.7 ± 1.24	95.7
-	SPCE	Tap water	30.0	31.4 ± 0.94	104.7
BES	AuNPs-SPCE	Tap water	30.0	31.0 ± 0.68	103.4

### Reproducibility of preparation of AuNPs-SPCE by BES and manually by lab operator

**Fig. 5:**
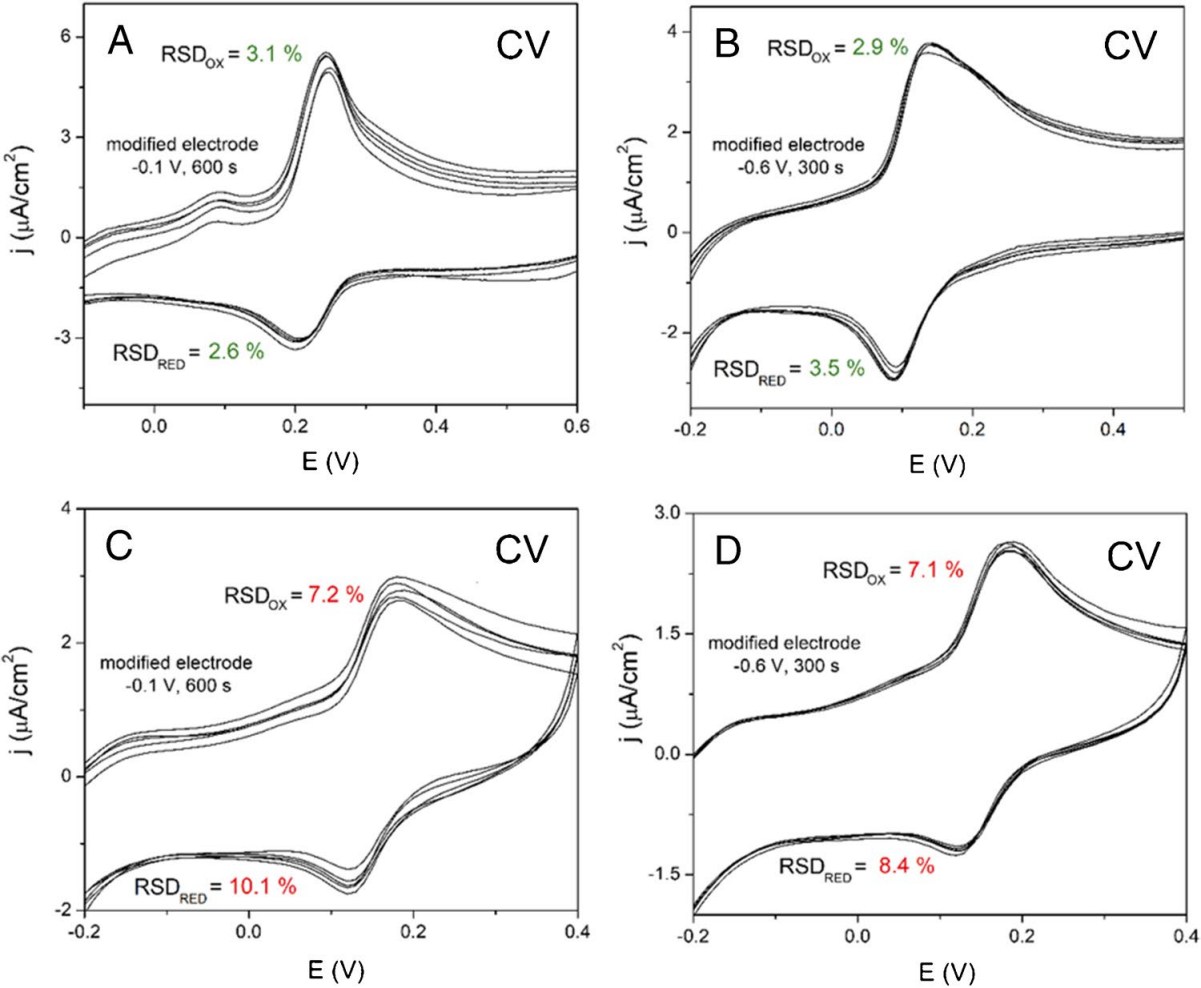
CV records of 50 μM DOP in BR pH 4.0 on five AuNP-SPCE sensors at different modification conditions, modified by BES (**A**, **B**) and manually by lab operator (**C**, **D**). Scan rate  100 mV/s

Besides plenty of benefits that comes from using sampler BES such as automatization, speeding up the process of analytical method development and lower consumption of chemicals and reagents, one of the most prominent benefits of BES, lies in elimination of systematic errors, especially personal errors from the side of laboratory operator. This was confirmed by our observations and experiments when the AuNPs-SPCE were prepared by the sampler BES and manually by a laboratory operator experienced in the field of analytical chemistry for several years. In this step, five AuNPs-SPCEs were modified at two various modifications conditions (*E*_DEP_ =  − 0.1 V, *t*_DEP_ = 600 s; and *E*_DEP_ =  − 0.6 V, *t*_DEP_ = 300 s), and then, 50 μM DOP in BR pH 4.0 was recorded by cyclic voltammetry using the modified sensors. The reproducibility of the preparation of the AuNP-SPCE sensors was achieved by assessing the voltammetric responses of 50 μM DOP modified by the BES and manually by laboratory operator. As was expected, the usage of the BES in the modification of the SPCE sensors led to the lower RSD at app. 3.0% (Fig. 5 A, B). On the other hand, in the case when the SPCE sensors were modified by the laboratory operator, the particular RSD value was attained at app. 7.0% and more (Fig. 5 C, D).

**Fig. 6:**
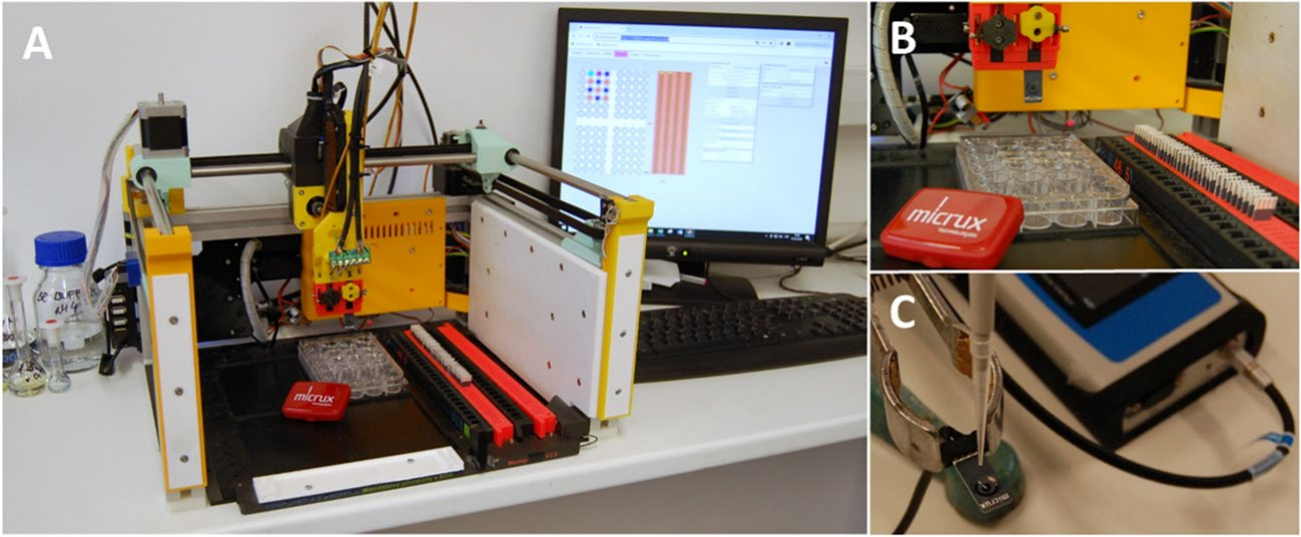
Photograph of the BES connected to PC with designed software (**A**, **B**) and manually set-up of the experimental measurement (**C**)

Another studied parameter was a stability of the deposition solution, especially the influence of vaporization of solvent and the possible increase/decrease of Au(III) concentration caused by the multiple electrodeposition AuNPs (15 modified SPCEs, *E*_DEP_ = − 0.1 V, *t*_DEP_ = 300 s). Fig. S26 shows that even after prepared fifteen AuNPs from the same deposition solution of 1 mM HAuCl_4_, the voltammetric response of 50 μM DOP on the 15th AuNPs-SPCE sensor was still comparable with only 3% decrease of the original voltammetric response. The results of this reproducibility study offer an unquestioning evidence about the advantages of the BES (Fig. 6) in the field of the modification of the electrode and then subsequent analytical measurements by the modified SPCE sensors.

## Conclusions

In this work, for the first time, the advanced BES was used for the fabrication of AuNP-SPCE sensor by the electrodeposition and the development of analytical protocol and followed quantification of PAR in the tap water samples by “spike-recovery” approach. Utilized BES offers a wide spectrum of benefits including automatization, acceleration of analytical procedure, elimination of systematic errors in the matter of laboratory operator at fabrication of modified electrode by electrodeposition or drop-casting and lower production of organic waste by performing analysis in small volume of analyzed solution. In terms of acceleration of analytical procedure by the BES, the overall analysis time represented app. half-length in comparison to the situation when analytical procedure was executed by highly skilled laboratory operator manually. In this context, it still remains unanswered how the data recorded by semi-skilled or fresh operator such as diploma student would look-like. Other considerable benefit arising from the BES usage leads to the favourable repeatability of the preparation of modified electrode, when values of RSD for five voltammetric responses of 50 μM DOP on the AuNPs-SPCE were at level of 3%. All mentioned advantages of the BES predetermine it for use in the various application fields such as high-throughput production of modified sensors, development and speeding up of the analytical methods or automated analytical system able to analyze large amounts of samples.

### Supplementary Information

Below is the link to the electronic supplementary material.

Supplementary file1 (DOC 47.2 MB)

## Data Availability

The authors declare that all data generated or analyzed during this study are included in this published article (and its supplementary information files). More needed information is available from the corresponding author upon reasonable request.
