# Supplementary material for: Proving the automatic benchtop electrochemical station for the development of dopamine and paracetamol sensors
Source: Mikrochim Acta. 2024 Jun 20;191(7):408. doi: 10.1007/s00604-024-06454-6 (PMC11186920; doi:10.1007/s00604-024-06454-6)
Supplement: Supplementary file 1 — Supplementary file1 (DOC 47.2 MB) [file 604_2024_6454_MOESM1_ESM.doc]

**SUPPLEMENTARY MATERIAL**

**PROVING OF THE AUTOMATIC BENCHTOP ELECTROCHEMICAL STATION FOR DEVELOPMENT OF DOPAMINE**

**AND PARACETAMOL SENSORS**

**Marek Haššo^1^, Jiří Kudr^2^, Jan Zítka^2^, Jan Šílený^2^, Pavel Švec^2^,**

**Ľubomír Švorc^1^, Ondřej Zítka^2,3*^,**

*^1^Institute of Analytical Chemistry, Faculty of Chemical and Food Technology, Slovak*

*University of Technology in Bratislava, Radlinského 9, Bratislava, SK-812 37, Slovakia*

*^2^Department of Chemistry and Biochemistry, Mendel University in Brno, Zemedelska 1, Brno CZ-613 00, Czech Republic*

*^3^Central European Institute of Technology, Brno University of Technology, Technicka 3058/10, Brno CZ-616 00, Czech Republic*

*E-mail: ondrej.zitka@mendelu.cz*

*
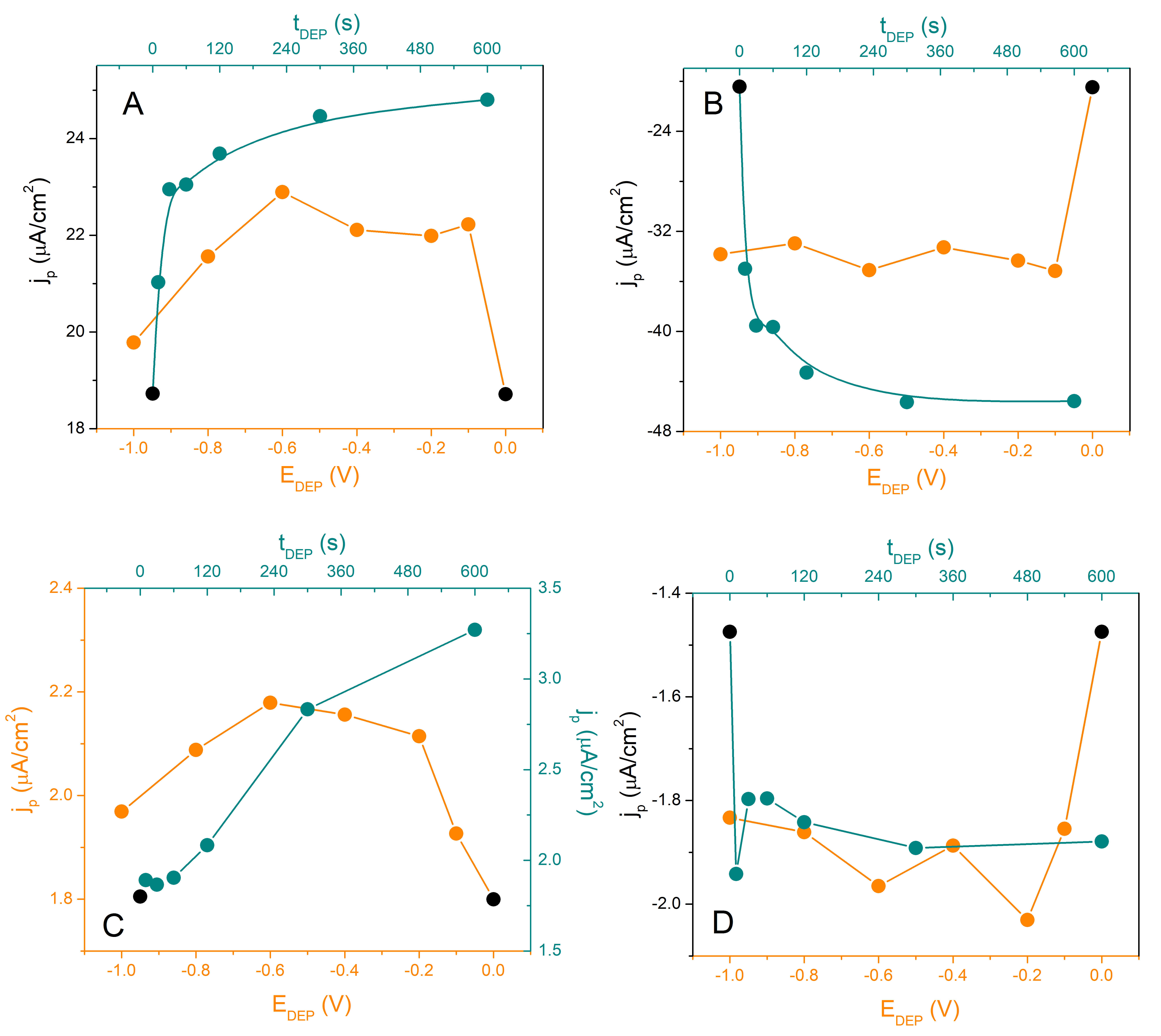
*

**Fig. S1 –** Dependences of oxidation and reduction peaks on deposition time j_p_ = f(*t*_DEP_) and deposition potential j_p_ = f (*E*_DEP_) for 1 mM [Ru(NH_3_)_6_]Cl_3_ in 0.1 M KCl **(A, B)** and 50 µM DOP in BR pH 4.0 **(C, D)**, black dots represent voltammetric responses registered on bare SPCE.

**
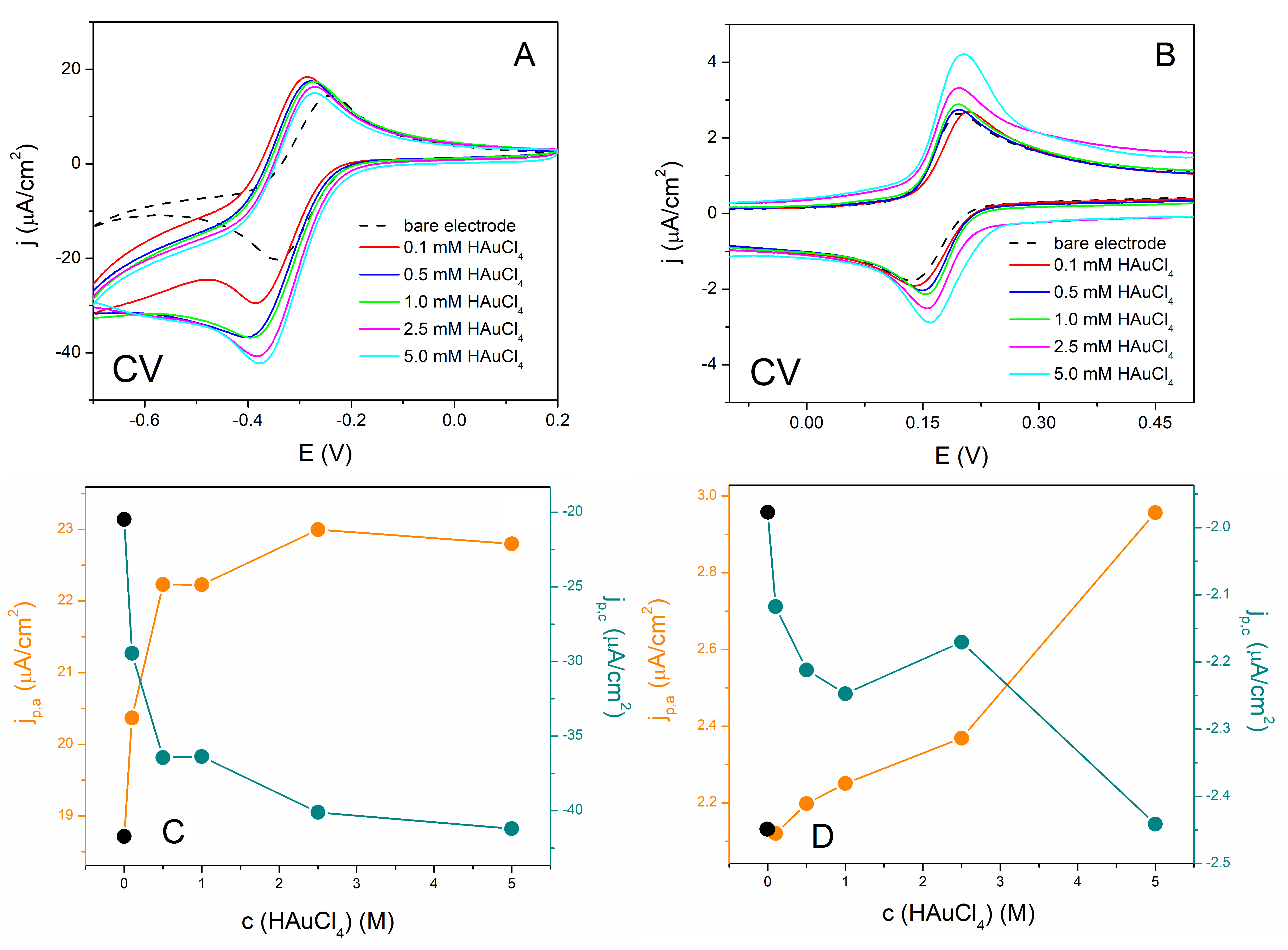
**

**Fig. S2 –** CV records of 1 mM [Ru(NH_3_)_6_]Cl_3_ in 0.1 M KCl **(A)** and 50 µM DOP in BR pH 4.0 **(B)** on SPCE and AuNPs-SPCE prepared from deposition solution at various concentrations of HAuCl_4_. Dependences of oxidation (orange line) and reduction (green line) peaks of 1 mM [Ru (NH_3_)_6_]Cl_3_ **(C)** and 50 µM DOP **(D)** on concentrations of HAuCl_4_, black dots represent voltammetric responses registered on bare SPCE.


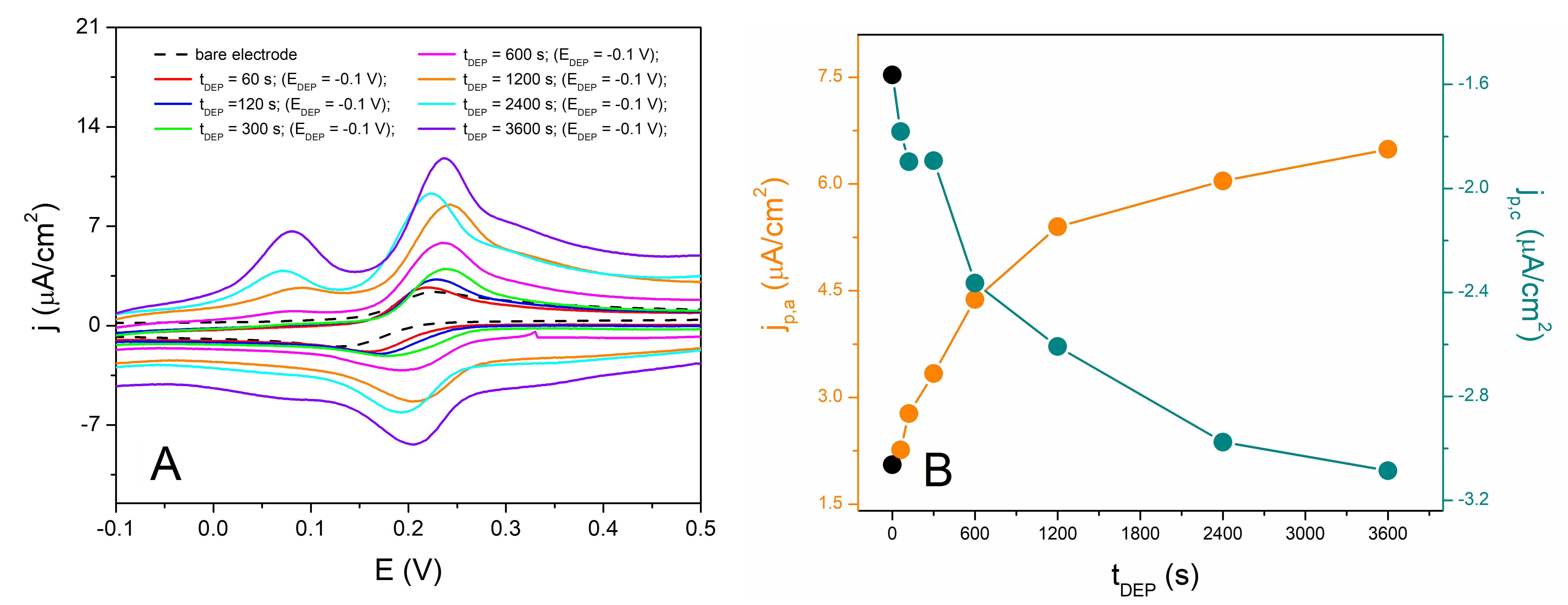


**Fig. S3 –** CV records of 50 µM DOP in BR pH 4.0 on SPCE and AuNPs-SPCE at spread range of *t*_DEP_ **(A)**, dependences of oxidation (orange line) and reduction (green line) peaks of 50 µM DOP on deposition time j_p_ = f(*t*_DEP_) **(B)**, black dots represent voltammetric responses registered on bare SPCE.


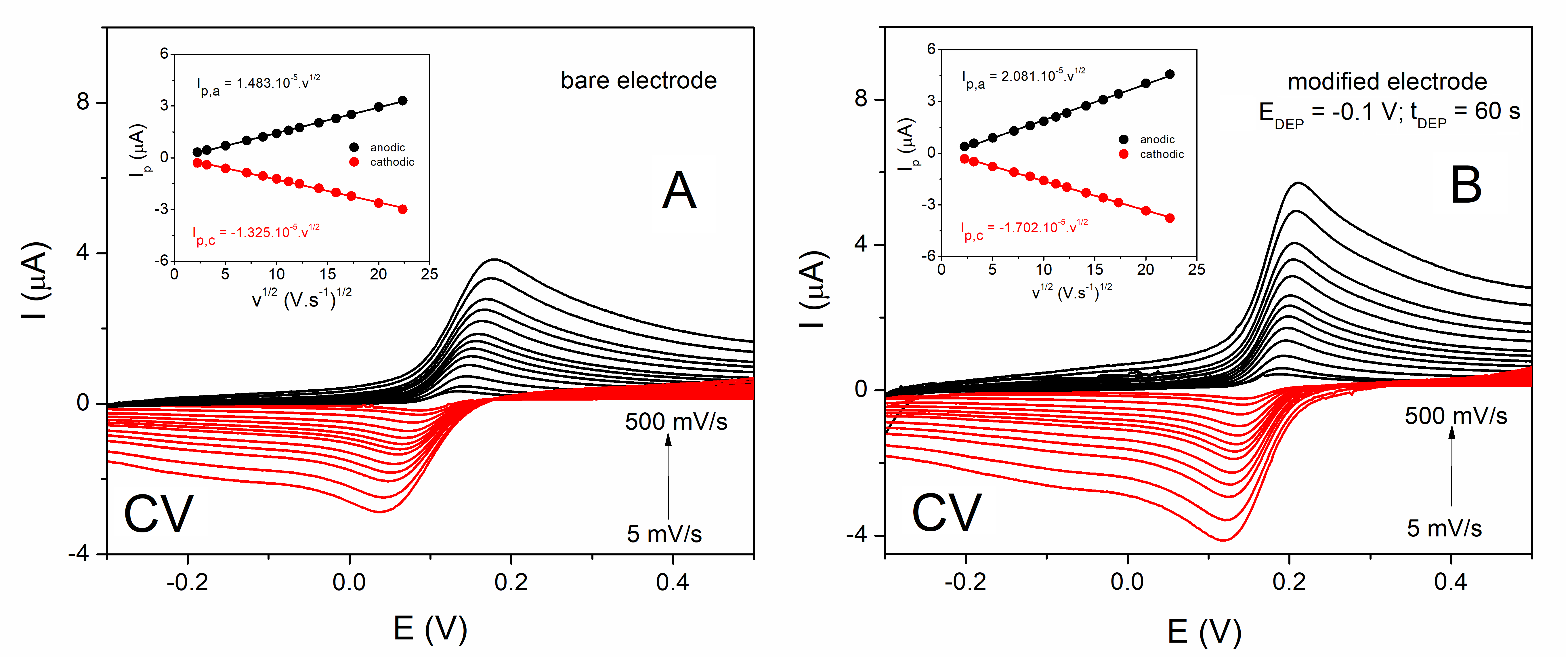


**Fig. S4 –** CV records of 50 µM DOP in BR pH 4.0 at various scan rate values *v* (5, 10, 25, 50, 75, 100, 125, 150, 200, 250, 300, 400, and 500 mV/s) and corresponding dependences between the anodic and cathodic *I*_p_ and *v*^1/2^ on bare SPCE (A) and AuNPs-SPCE (B).


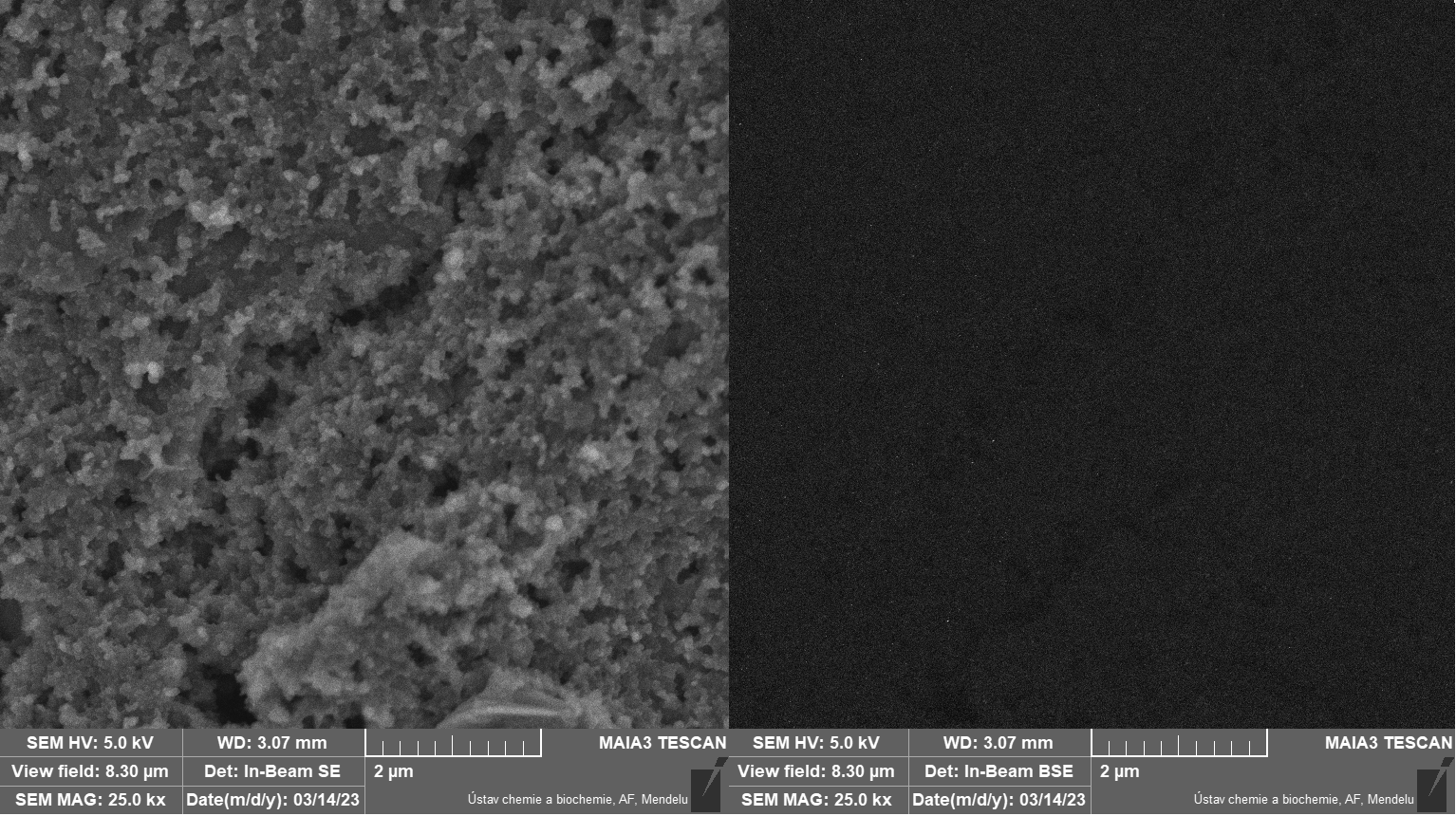


**Fig. S5 –** SEM images of bare SPCE at scanning of secondary electrons (SE) **(Left)** and scanning of backscattered electrons (BSE) **(Right)**, 25 kx magnification.


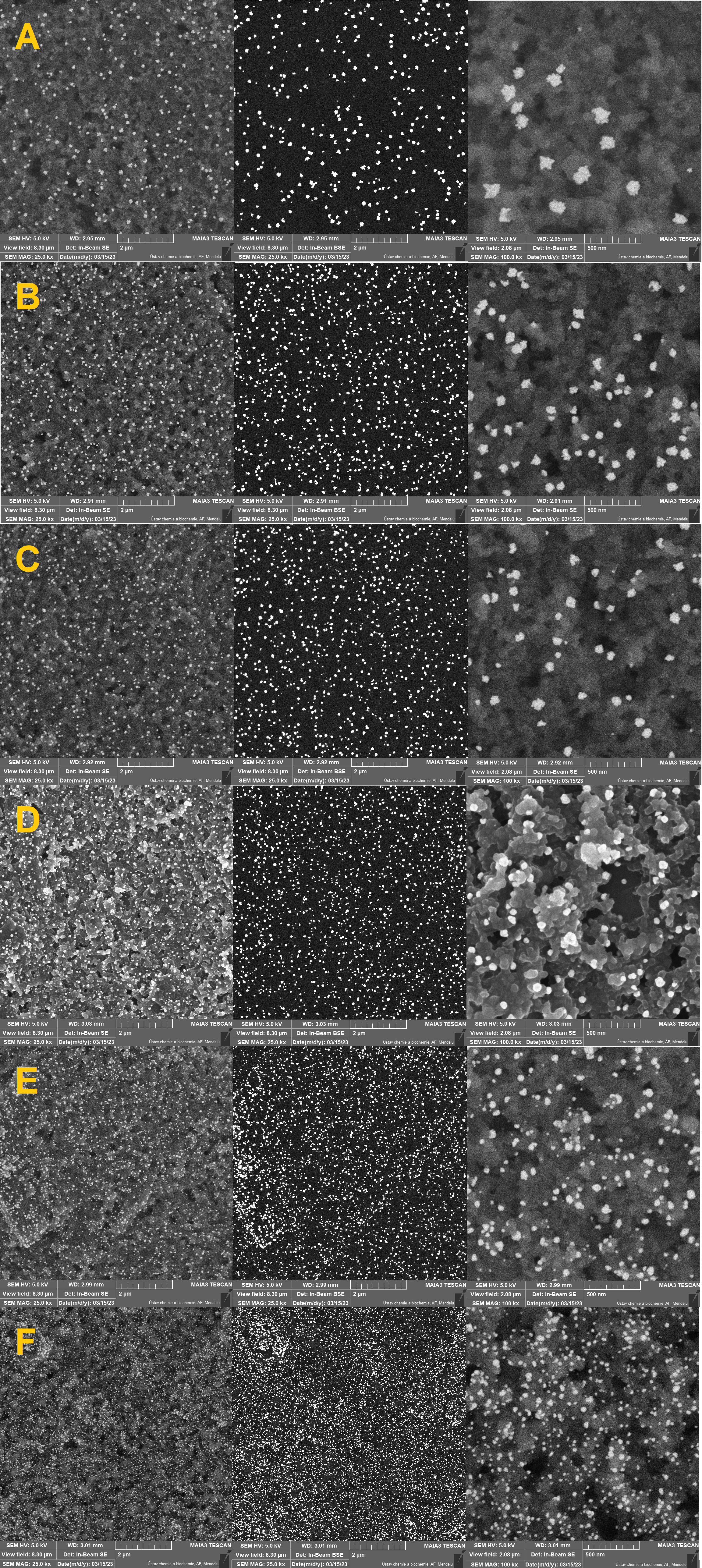


**Fig. S6 –** SEM images of AuNPs-SPCE prepared at different *E*_DEP_ = –0.1 V **(A)**; –0.2 V **(B)**;
–0.4 V **(C)**; –0.6 V **(D)**; –0.8 V **(E)**; –1.0 V **(F)**. Images taken at 25kx magnification in SE mode **(Left)** and BSE mode **(Middle)**, Images taken at 100kx magnification in SE mode **(Right)**.


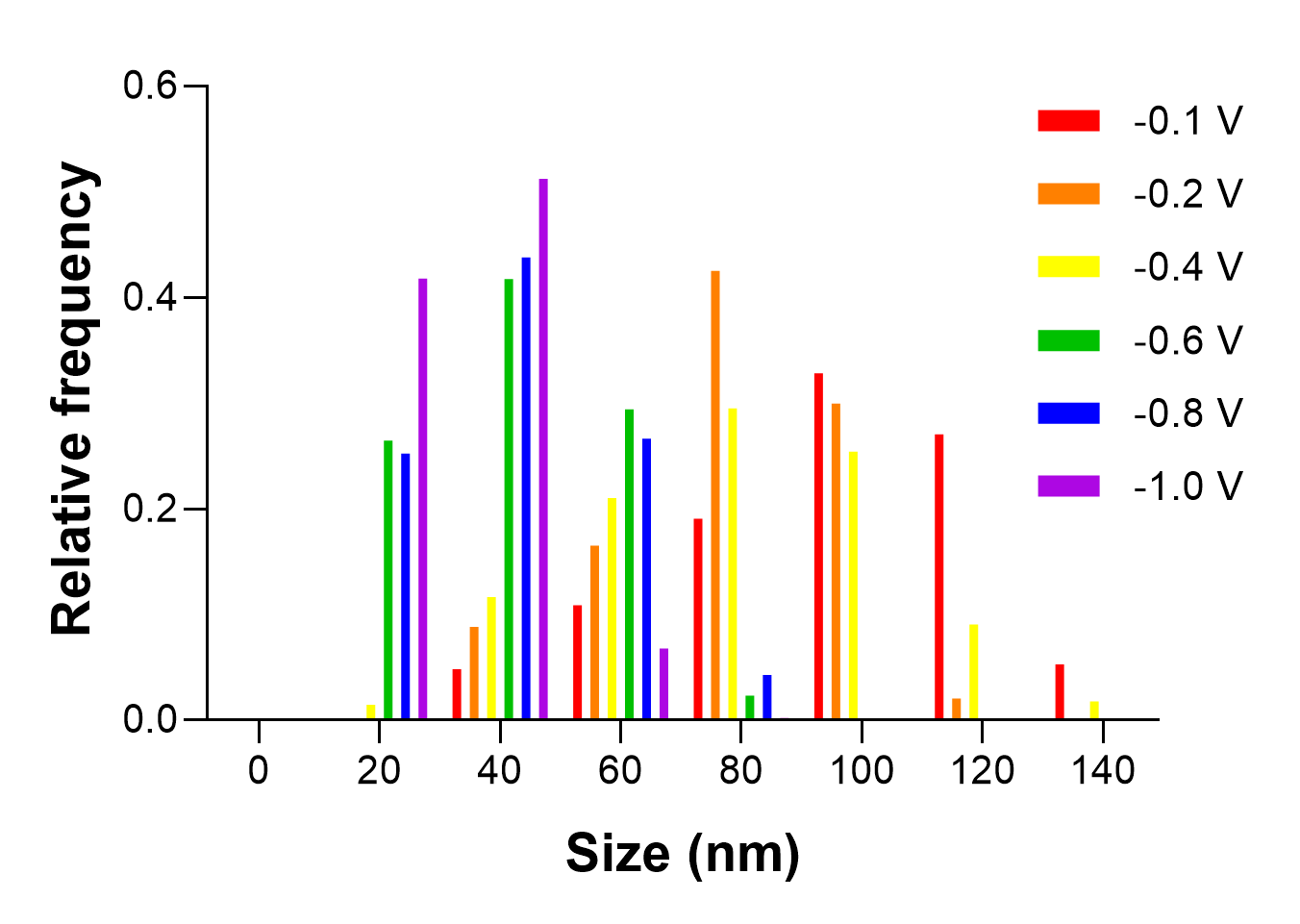


**Fig. S7 –** Histogram represents relative frequency of Au nanoparticles of various size prepared at individual deposition potential *E*_DEP_ = –0.1 V; –0.2 V; –0.4 V; –0.6 V; –0.8 V;
–1.0 V and *t*_DEP_ = 60 s, in the presence of 1 mM HAuCl_4_ in 0.1 M H_2_SO_4_.


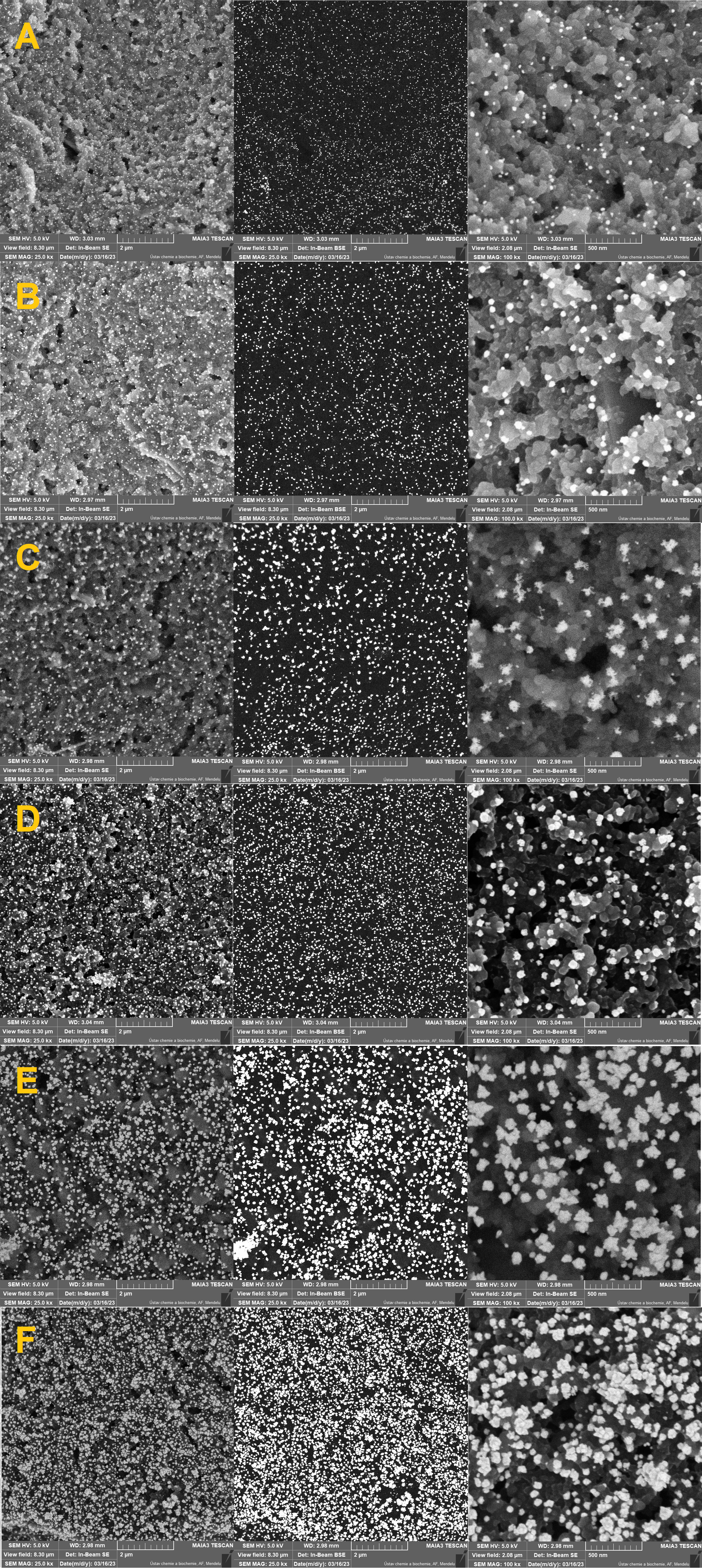


**Fig. S8 –** SEM images of AuNPs-SPCE prepared at different *t*_DEP_ = 10 s **(A)**; 30 s **(B)**; 60 s **(C)**; 120 s **(D)**; 300 s **(E)**; 600 s **(F)**. Images taken at 25kx magnification in SE mode **(Left)** and BSE mode **(Middle)**, Images taken at 100kx magnification in SE mode **(Right)**.


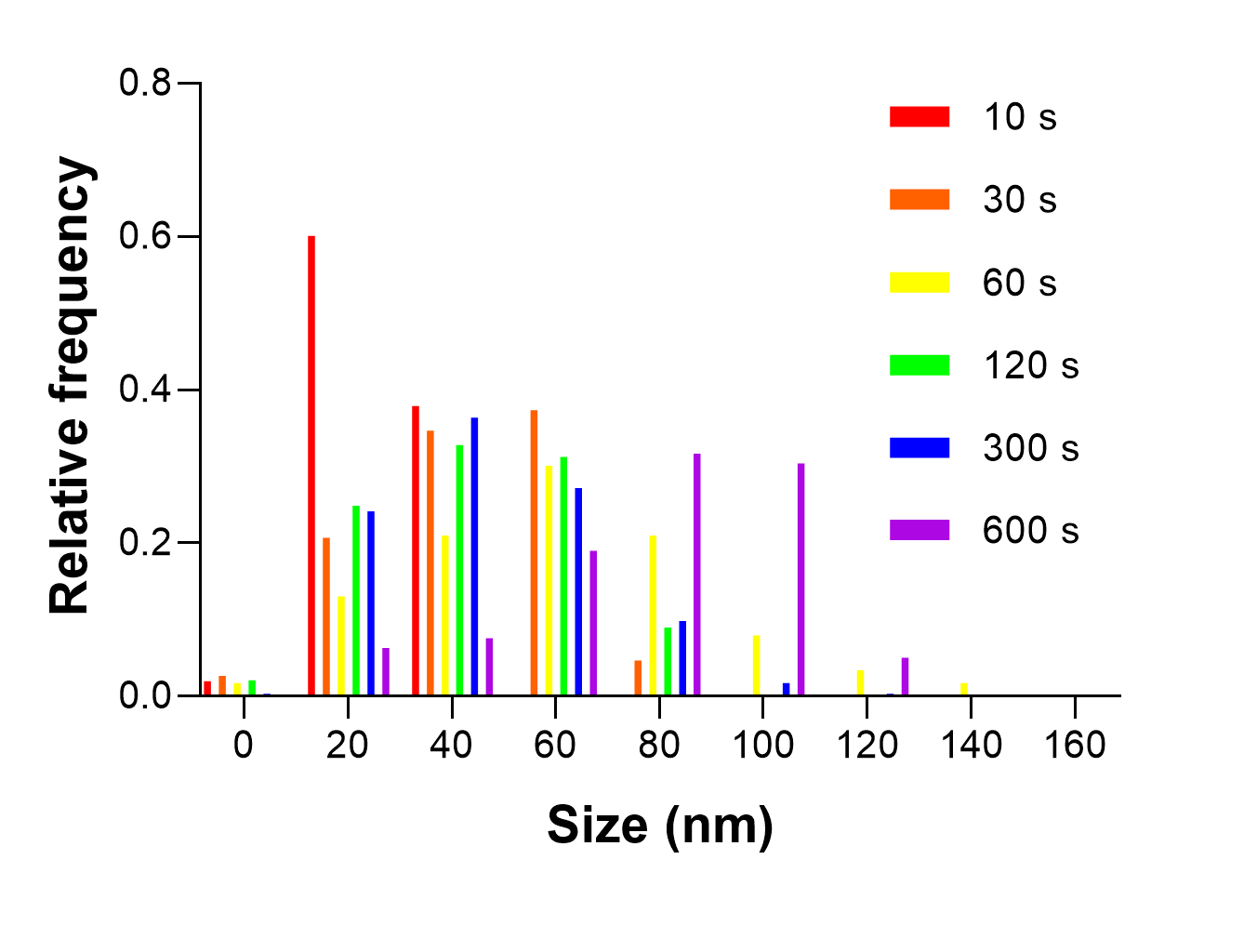


**Fig. S9 –** Histogram represents relative frequency of Au nanoparticles of various size prepared at individual deposition time *t*_DEP_ = 10 s; 30 s; 60 s; 120 s; 300 s;
600 s and *E*_DEP_ = –0.1 V, in the presence of 1 mM HAuCl_4_ in 0.1 M H_2_SO_4_.


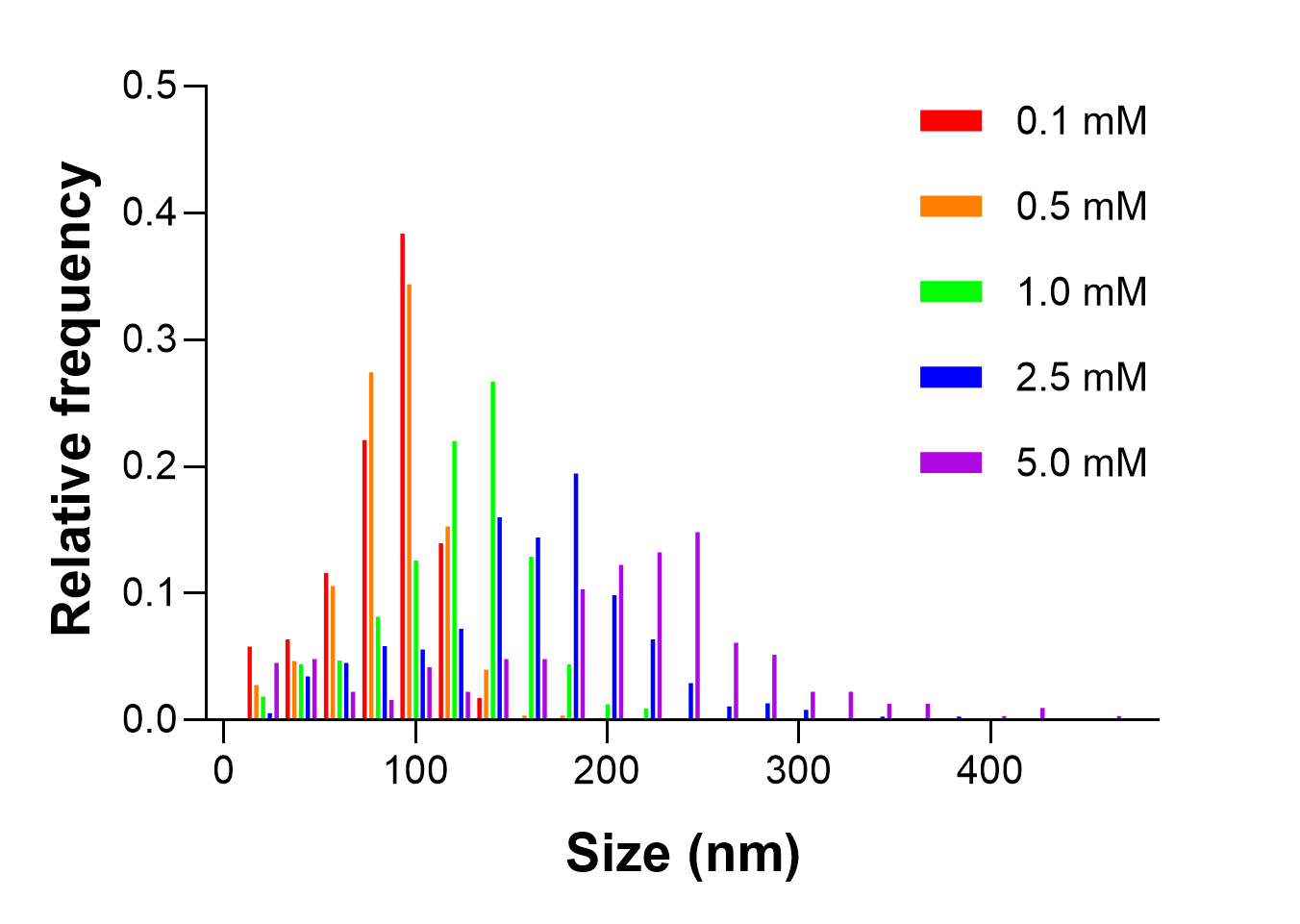


**Fig. S10 –** Histogram represents relative frequency of Au nanoparticles of various size prepared at individual deposition time *t*_DEP_ = 10 s; 30 s; 60 s; 120 s; 300 s;
600 s and *E*_DEP_ = –0.1 V, in the presence of 1 mM HAuCl_4_ in 0.1 M H_2_SO_4_.


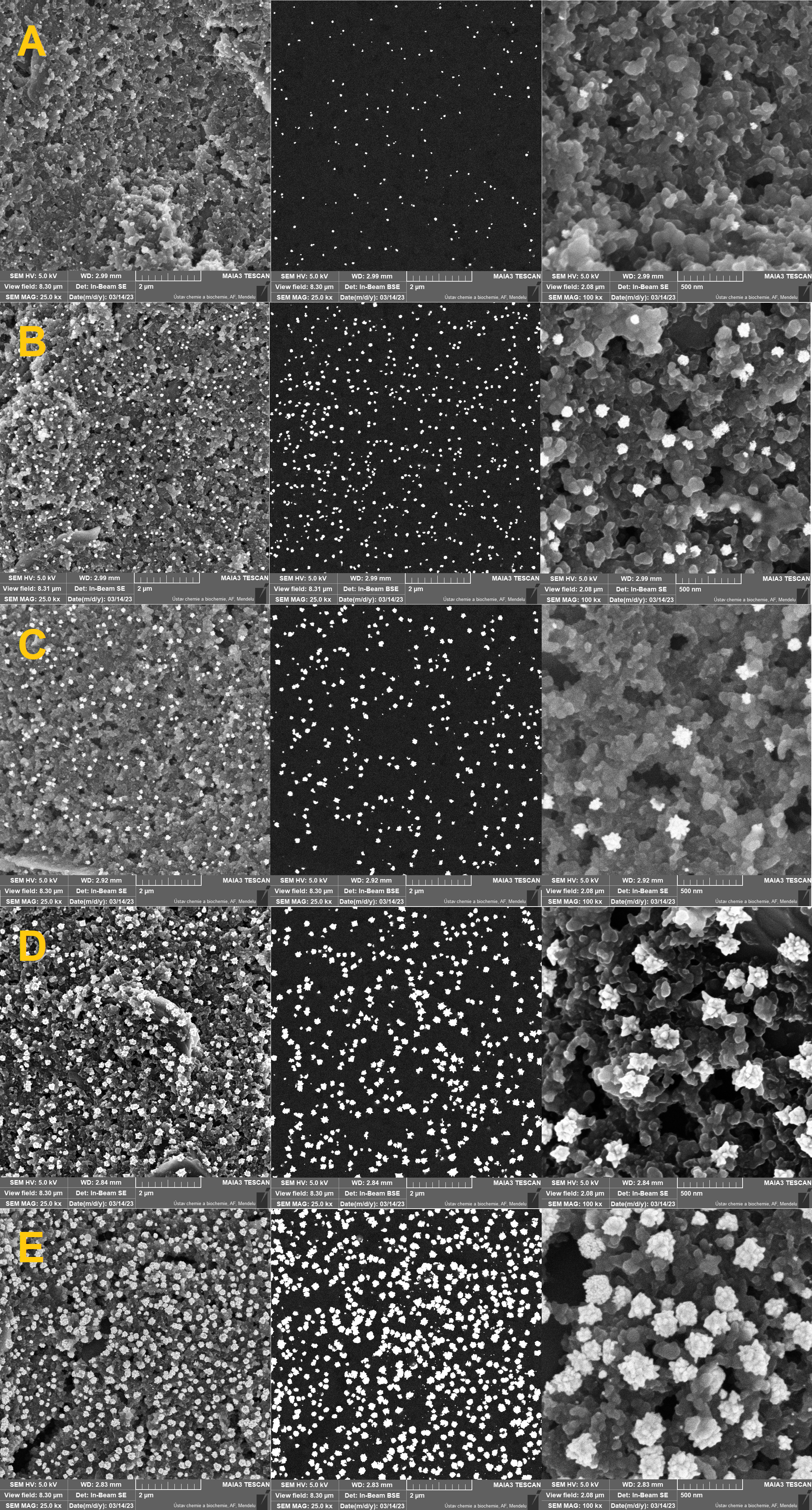


**Fig. S11 –** SEM images of AuNPs-SPCE prepared in HAuCl_4_ with different concentrations: 0.1 M **(A)**; 0.5 M **(B)**; 1.0 M **(C)**; 2.5 M **(D)**; 5.0 M **(E)**. Images taken at 25kx magnification in SE mode **(Left)** and BSE mode **(Middle)**, Images taken at 100kx magnification in SE mode **(Right)**.


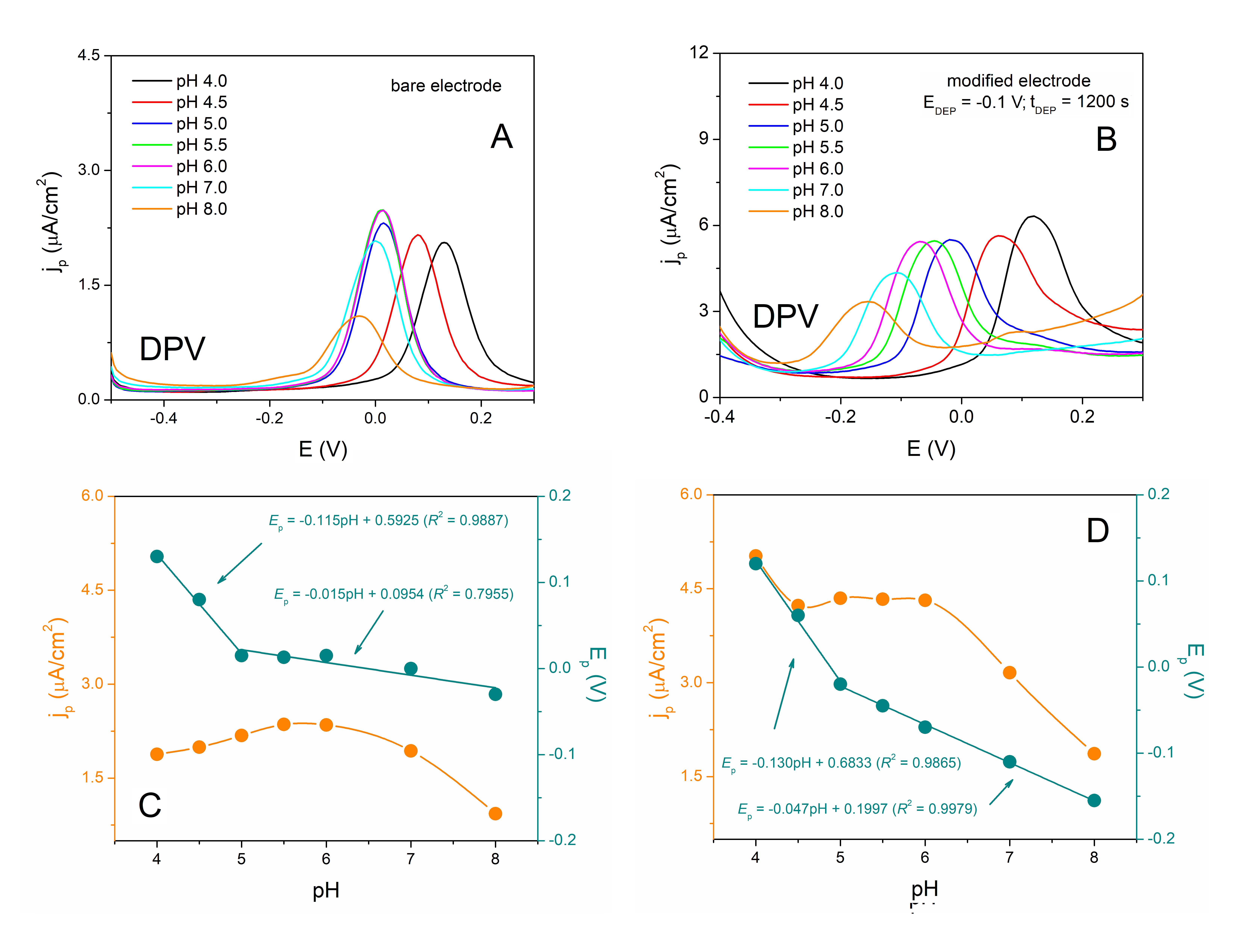


**Fig. S12 –** DP voltammograms of 50 µM DOP in BR buffer at different pH values (4.0 – 8.0) on bare SPCE **(A)** and AuNPs-SPCE **(B)**. Dependence of current density (j_p_) and peak potential (*E*_p_) of 50 µM DOP on pH of BR buffer, recorded on bare SPCE **(C)** and modified SPCE **(D)**. Pulse parameters: pulse height 100 mV, pulse time 100 ms and interval time 0.5 s.


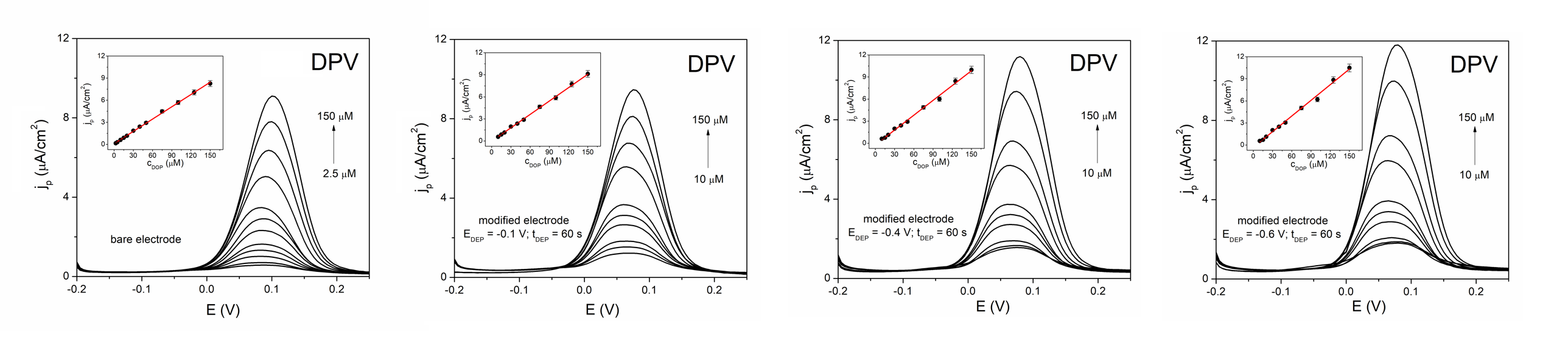


**Fig. S13 –** DP voltammograms of calibration solutions of DOP in BR pH 4.0 in the concentration ranges from 2.5 µM to 150 µM (bare SPCE) and 10 µM to 150 µM (AuNPs-SPCE at various conditions of *E*_DEP_) and their corresponding calibration curves (insets). Pulse parameters: pulse height 100 mV, pulse time 100 ms and interval time 0.5 s.

**Table T1 -** Analytical parameters for the determination of DOP on bare SPCE and AuNPs-SPCE at various *E*_DEP_ (*n* = 3).

| **Parameter** | **Bare electrode** | ***E*_DEP_ = −0.1 V** | ***E*_DEP_ = −0.2 V** | ***E*_DEP_ = −0.4 V** | ***E*_DEP_ = −0.6 V** | ***E*_DEP_ = −0.8 V** | ***E*_DEP_ = −1.0 V** |
| --- | --- | --- | --- | --- | --- | --- | --- |
| Intercept (µA/cm^2^) | 0.105 ± 0.046 | −0.014 ± 0.060 | −0.100 ± 0.112 | −0.214 ± 0.123 | −0.301 ± 0.138 | −0.335 ± 0.143 | −0.332 ± 0.164 |
| Slope (µA/cm^2^.µM) | 0.056 ± 0.001 | 0.061 ± 0.001 | 0.063 ± 0.002 | 0.067 ± 0.002 | 0.071 ± 0.002 | 0.071 ± 0.002 | 0.072 ± 0.002 |
| LCR (µM) | 2.5 – 150 | 10 – 150 | 10 – 150 | 10 – 150 | 10 – 150 | 10 – 150 | 10 – 150 |
| *R*^2^ | 0.9985 | 0.9985 | 0.9952 | 0.9950 | 0.9943 | 0.9938 | 0.9921 |
| LOD (µM) | 2.5 | 3.0 | 5.4 | 5.5 | 5.9 | 6.1 | 6.9 |

**
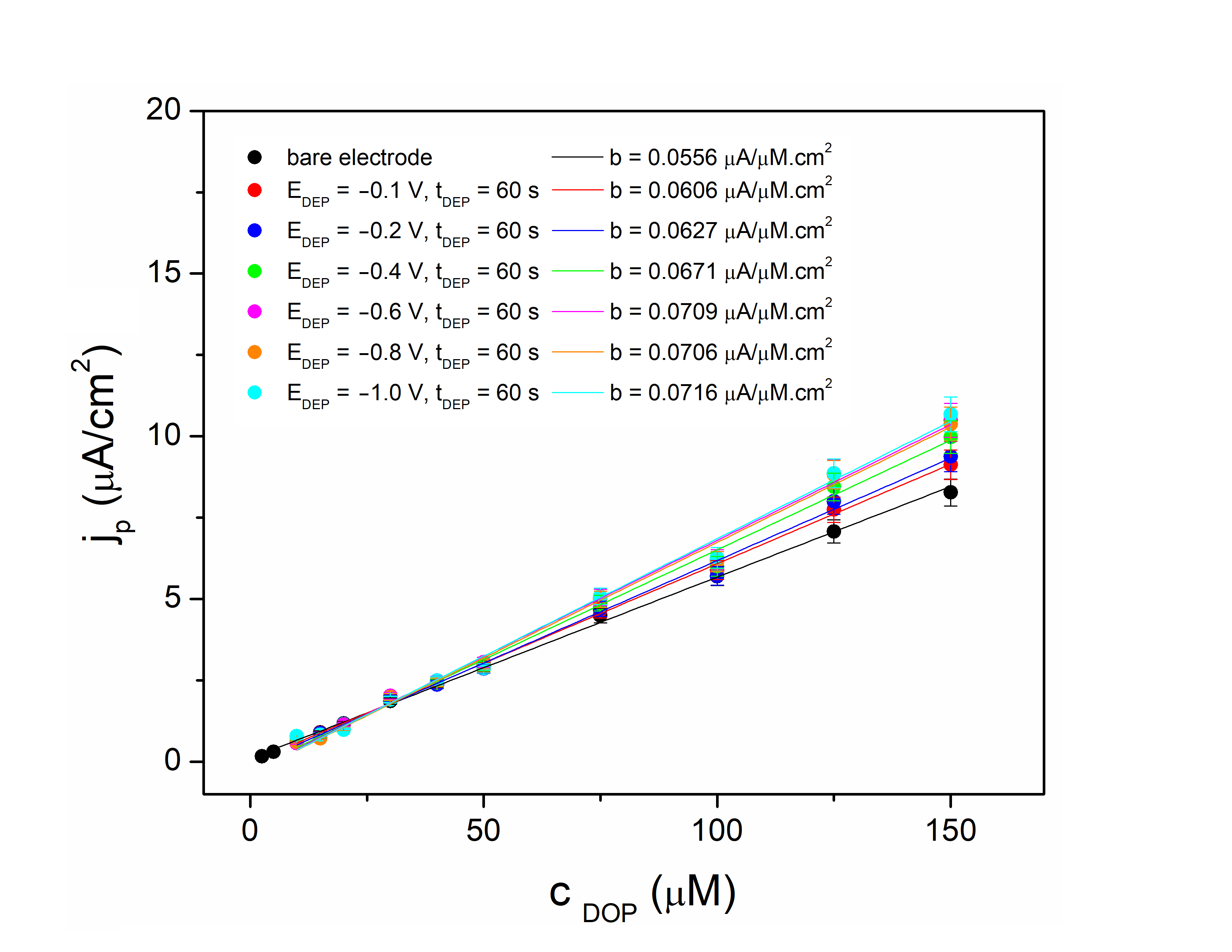
**

**Fig. S14 –** Comparison of constructed calibration curves of DOP on bare and AuNPs-SPCE at various conditions of *E*_DEP_ and their corresponding sensitivity to DOP.


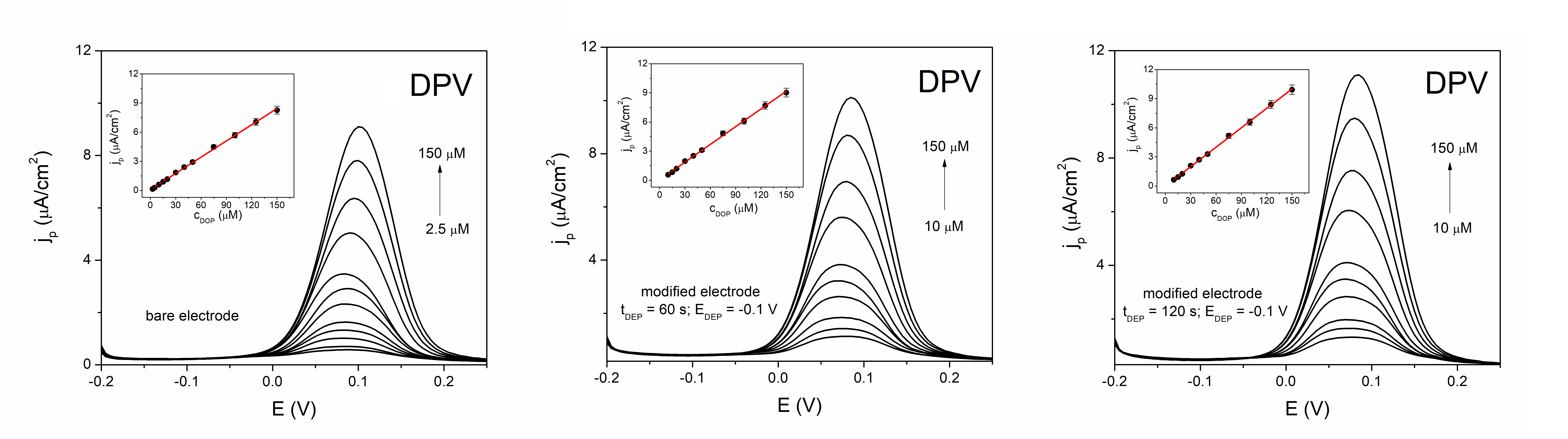


**Fig. S15 –** DP voltammograms of calibration solutions of DOP in BR pH 4.0 in the concentration ranges from 2.5 µM to 150 µM (bare SPCE) and 10 µM to 150 µM (AuNPs-SPCE at various conditions of *t*_DEP_) and their corresponding calibration curves (insets). Pulse parameters: pulse height 100 mV, pulse time 100 ms and interval time 0.5 s.

**Table T2 -** Analytical parameters for the determination of DOP on bare SPCE and AuNPs-SPCE at various *t*_DEP_ (*n* = 3).

| **Parameter** | **Bare electrode** | ***t*_DEP_ = 60 s** | ***t*_DEP_ = 120 s** | ***t*_DEP_ = 300 s** | ***t*_DEP_ = 600 s** | ***t*_DEP_ = 1200 s** |
| --- | --- | --- | --- | --- | --- | --- |
| Intercept (µA/cm^2^) | 0.105 ± 0.040 | 0.044 ± 0.063 | 0.004 ± 0.050 | −0.019 ± 0.056 | −0.267 ± 0.054 | −0.184 ± 0.053 |
| Slope (µA/cm^2^.µM) | 0.056 ± 0.001 | 0.061 ± 0.001 | 0.067 ± 0.001 | 0.063 ± 0.001 | 0.064 ± 0.001 | 0.057 ± 0.001 |
| LCR (µM) | 2.5 – 150 | 10 – 150 | 10 – 150 | 10 – 150 | 10 – 150 | 10 – 150 |
| *R*^2^ | 0.9987 | 0.9984 | 0.9991 | 0.9988 | 0.9989 | 0.9987 |
| LOD (µM) | 2.2 | 3.1 | 2.3 | 2.7 | 2.6 | 2.8 |

**
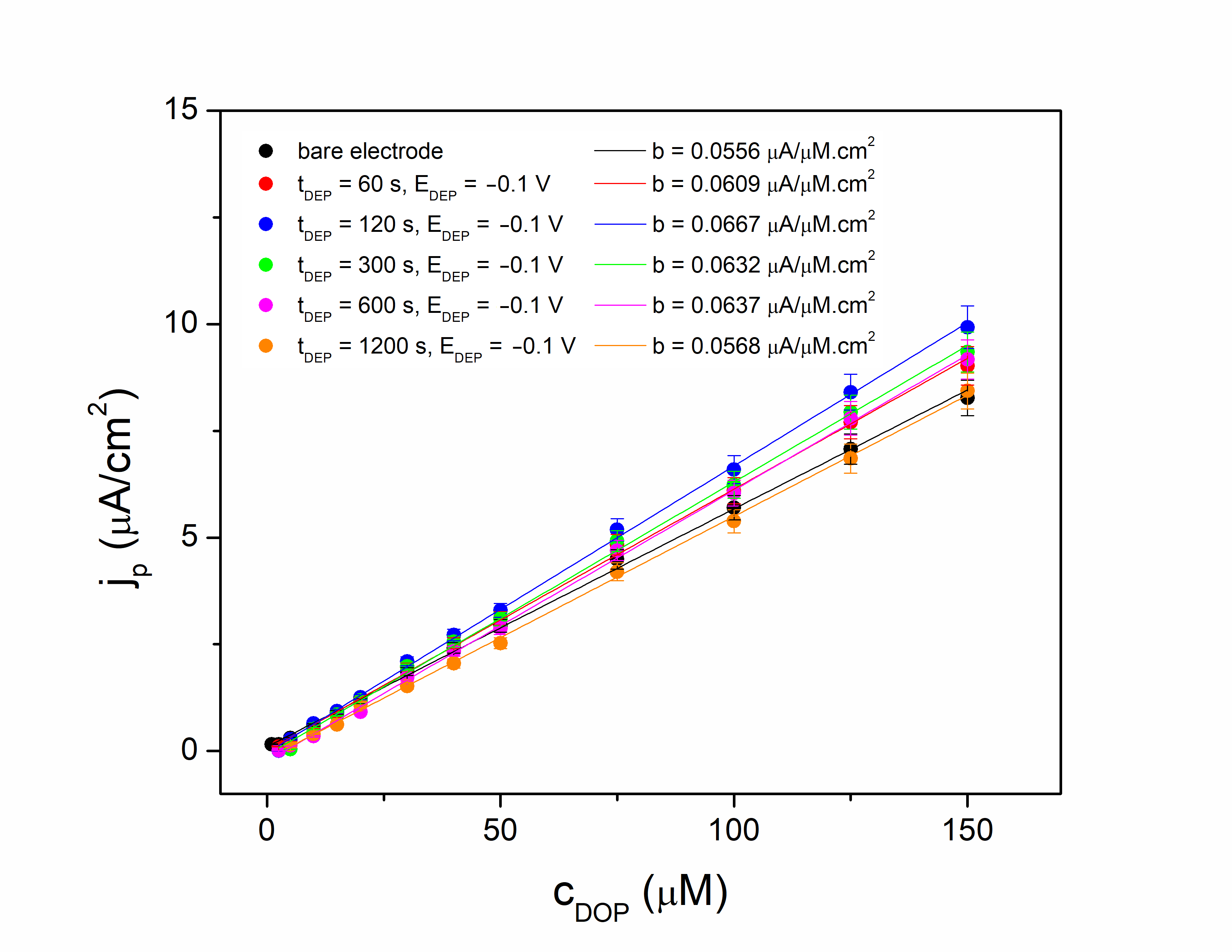
**

**Fig. S16 –** Comparison of constructed calibration curves of DOP on bare and AuNPs-SPCE at various conditions of *t*_DEP_ and their corresponding sensitivity to DOP.

**
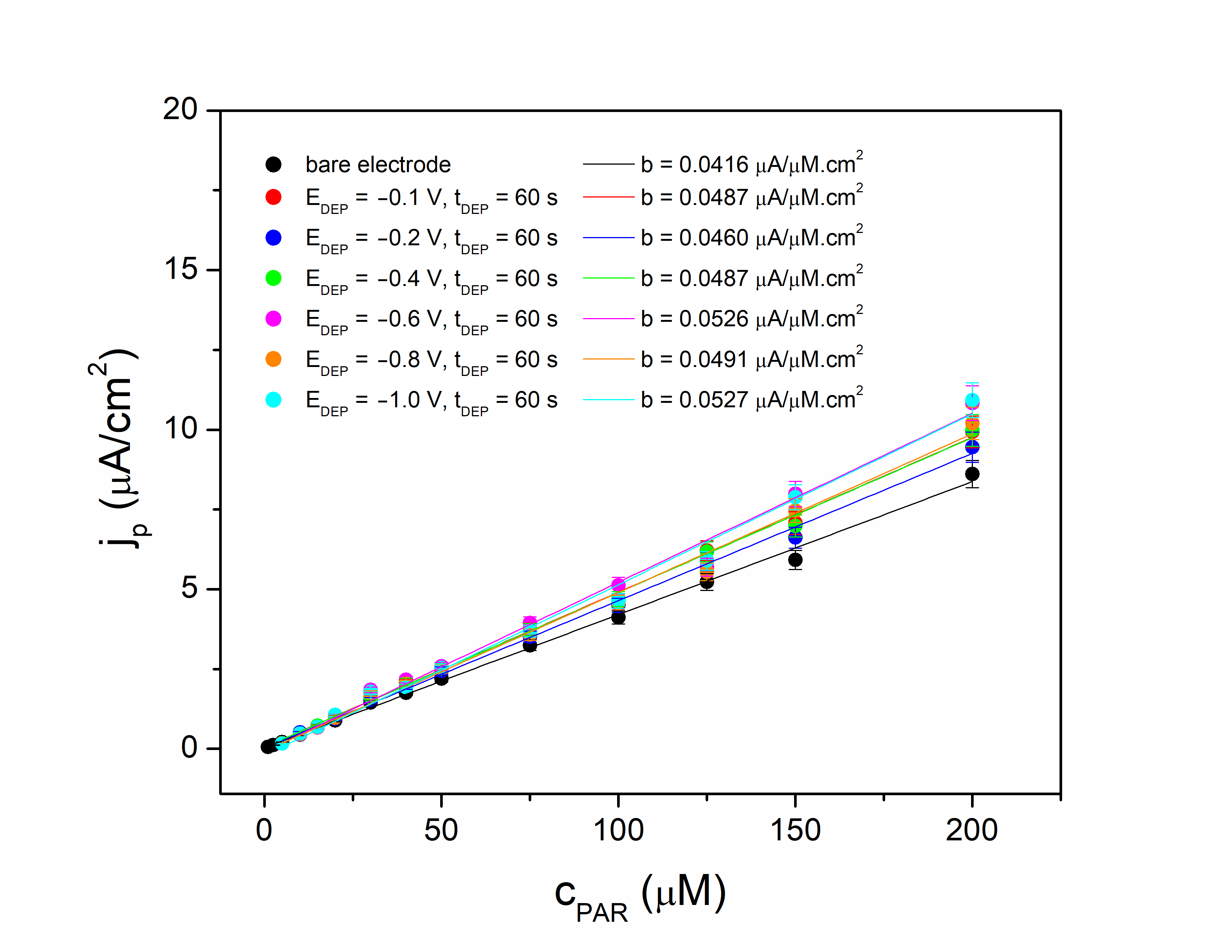
**

**Fig. S17 –** Comparison of constructed calibration curves of PAR on bare and AuNPs-SPCE at various conditions of *E*_DEP_ and their corresponding sensitivity to PAR.

**
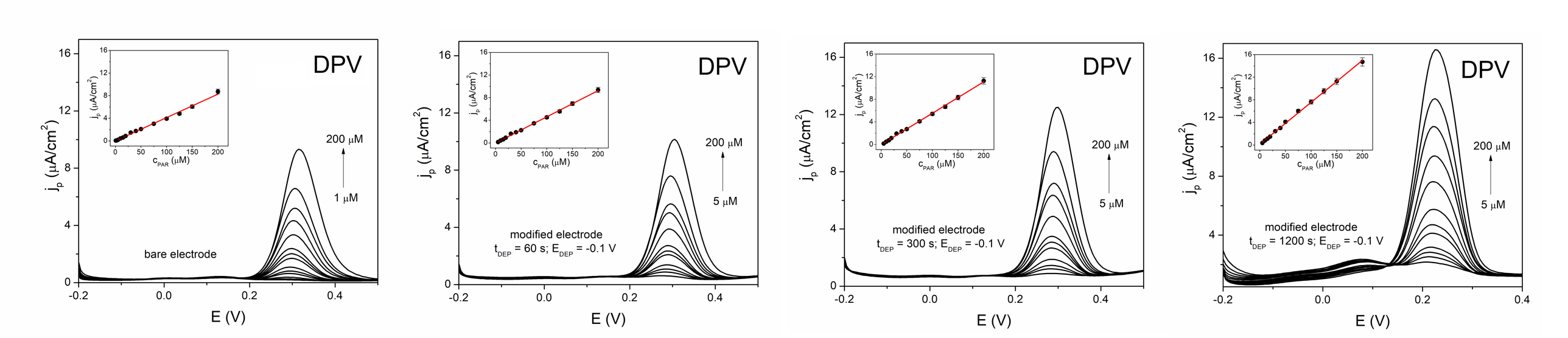
**

**Fig. S18 –** DP voltammograms of calibration solutions of PAR in BR pH 4.0 in the concentration ranges from 1 µM to 200 µM (bare SPCE) and 5 µM to 200 µM (AuNPs-SPCE at various conditions of *t*_DEP_) and their corresponding calibration curves (insets). Pulse parameters: pulse height 100 mV, pulse time 100 ms and interval time 0.5 s.

**Table T3 -** Analytical parameters for the determination of PAR on bare SPCE and AuNPs-SPCE at various *t*_DEP_ (*n* = 3).

| **Parameter** | **Bare electrode** | **­­­t_DEP_ = 60 s** | **­­­t_DEP_ = 120 s** | **­­­t_DEP_ = 300 s** | **­­­t_DEP_ = 600 s** | **­­­t_DEP_ = 1200 s** |
| --- | --- | --- | --- | --- | --- | --- |
| Intercept (µA/cm^2^) | 0.003 ± 0.054 | −0.007 ± 0.059 | −0.012 ± 0.062 | −0.018 ± 0.065 | 0.039 ± 0.078 | 0.144 ± 0.080 |
| Slope (µA/cm^2^.µM) | 0.041 ± 0.001 | 0.045 ± 0.001 | 0.053 ± 0.001 | 0.056 ± 0.001 | 0.063 ± 0.001 | 0.074 ± 0.001 |
| LCR (µM) | 1 – 200 | 5 – 200 | 5 – 200 | 5 – 200 | 5 – 200 | 5 – 200 |
| *R*^2^ | 0.9927 | 0.9974 | 0.9925 | 0.9982 | 0.9980 | 0.9985 |
| LOD (µM) | 3.9 | 3.7 | 3.5 | 3.5 | 3.8 | 3.2 |

**
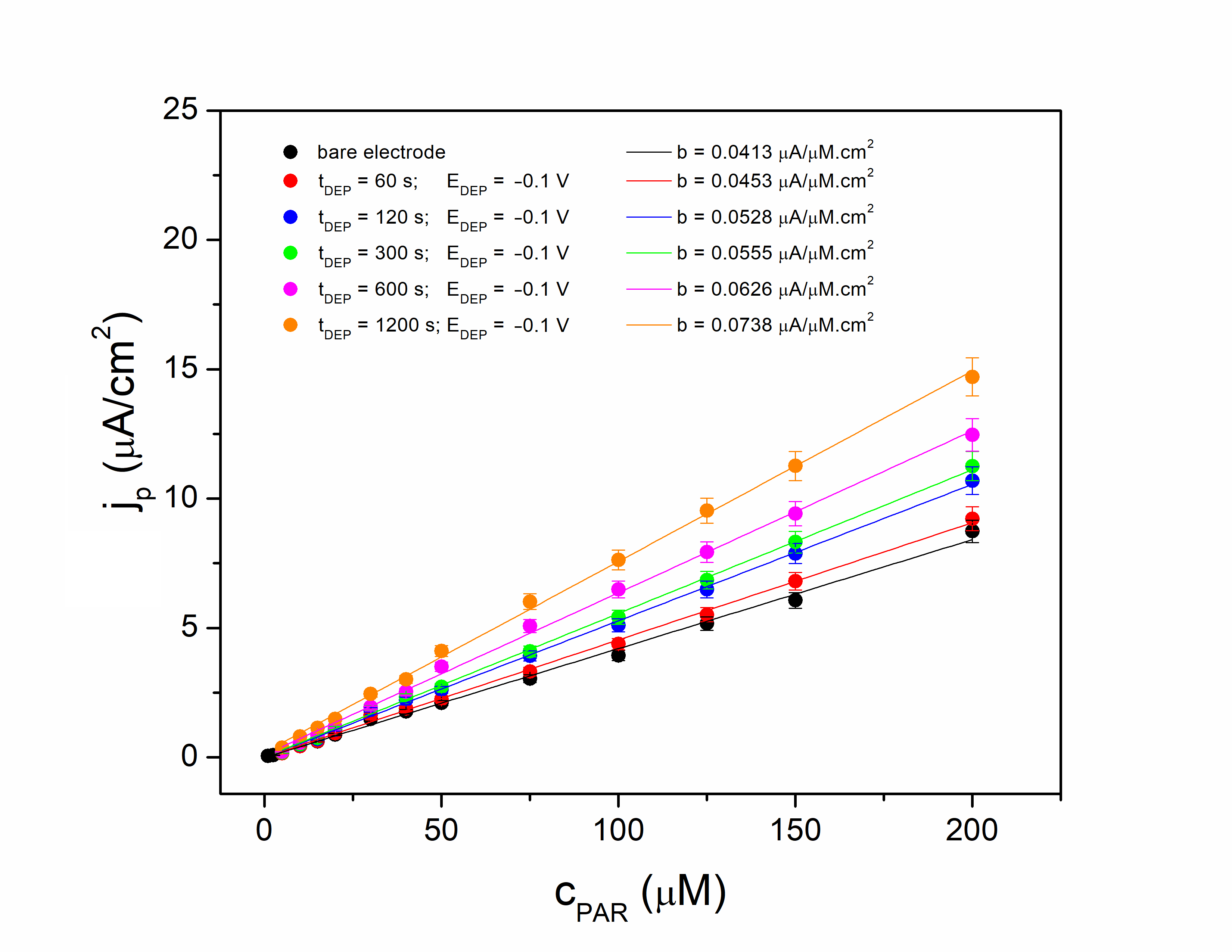
**

**Fig. S19 –** Comparison of constructed calibration curves of PAR on bare and AuNPs-SPCE at various conditions of *t*_DEP_ and their corresponding sensitivity to PAR.

**Table T4 –** Comparison of basic experimental and validation parameters of previously published electrochemical methods for the determination of DOP and PAR

| **Working electrode** | **Analyte** | **Detection technique** | **Supporting electrolyte** | ***E*_p_ (V)** | **LCR  (μM)** | **LOD  (μM)** | **Analyzed samples** | **Ref.** |
| --- | --- | --- | --- | --- | --- | --- | --- | --- |
| δ-MnO_2_/S@g-C_3_N_4_-SPCE | DOP | LSV | 0.1 M PBS pH 7.0 | +0.25 | 3 – 125 | 1.12 | – | [47] |
| LI-EPPG-SPE | DOP | DPV | 0.1 M PBS pH 4.0 | +0.27 | 0.34 – 1.60 | 0.08 | – | [48] |
| AuNPs-SPCE | DOP | LSV | 0.1 M PBS pH 7.4 | +0.08 | 2 – 100 | 0.20 | Tablet | [49] |
| WO_3_-SPE | DOP | SWV | 0.1 M PBS pH 7.0 | +0.51 | 1 – 250 | 0.87 | Urine | [50] |
| PDbS–rGO-SPCE | DOP | LSV | 0.05 M PBS pH 7.0 | +0.13 | 0.1 – 300 | 0.13 | Brain Tissue | [51] |
| ERGNRs/SPCE | DOP | DPV | 0.1 M PBS pH 7.0 | +0.065 | 0.50 – 300 | 0.15 | Urine | [52] |
| **AuNPs-SPCE** | **DOP** | **DPV** | **BRBS pH 4.0** | **+0.10** | **5 – 150** | **2.3** | **Tap water** | **This work** |
| NiNPS-SDS/CS-SPE | PAR | DPV | 0.1 M PBS pH 7.0 | +0.28 | 1 – 2000 | 0.04 | Tablet | [53] |
| CNFs-SPCE | PAR | DPAdSV | 0.1 M H_2_SO_4_ | +0.5 | 0.002 – 0.05  0.1 – 2.0 | 0.00054 | River and sea water | [54] |
| CNFs-SPE | PAR | DPV | ABS pH 5.5 | +0.35 | 0.002 – 0.034 mg/L | 0.0002 | Tap water | [55] |
| MWCNTs-COOH-SPCE | PAR | DPAdSV | ABS pH 4.0 | +0.33 | 0.005 – 5 | 0.001 | Wastewater | [56] |
| SDS-aSPCE | PAR | DPAdSV | ABS pH 4. | +0.26 | 0.05 – 20 | 0.015 | River water and serum | [57] |
| MXene-SPE | PAR | DPV | 0.1 M H_2_SO_4_ | +0.45 | 0.25 – 500  600 – 2000 | 0.048 | Tablet | [58] |
| **AuNPs-SPCE** | **PAR** | **DPV** | **BRBS pH 4.0** | **+0.22** | **5 – 200** | **3.2** | **Tap water** | **This work** |

Abbreviations:

δ-MnO_2_/S@g-C_3_N_4_-SPCE- – screen printed carbon electrode modified by composite of manganese dioxide with sulfur-doped graphitic carbon nitride

ABS – acetate buffer solution

AuNPs-SPCE – screen printed carbon electrode modified by gold nanoparticles

BRBS – Britton-Robinson buffer solution

CNFs-SPCE – screen printed carbon electrode modified by carbon-nanofibers

CNFs-SPE – screen printed electrode modified by carbon-nanofibers

DOP – dopamine

DPV – differential-pulse voltammetry

DPAdsSV – differential-pulse adsorptive stripping voltammetry

ERGNRs/SPCE – screen printed carbon electrode modified by electrochemically reduced graphene nanoribbons

LCR – linear concentration range

LI-EPPG-SPE - laser-induced edge-plane pyrolitic graphite screen-printed electrode

LOD – limit of detection

LSV – linear-sweep voltammetry

MWCNTs-COOH-SPCE – screen printed carbon electrode modified by carboxyl functionalized multiwalled carbon nanotubes

MXene-SPE – screen printed electrode modified by MXene

NiNPS-SDS/CS-SPE – screen printed electrode modified by chitosan-coated nickel nanoparticles with anion surfactant sodium dodecyl sulphate

PAR – paracetamol

PBS – phosphate buffer solution

PDbS–rGO/SPCE – screen printed carbon electrode modified by poly(DMAEMA-b-styrene with reduced graphene oxide

SDS-aSPCE – electrochemically activated screen printed carbon electrode modified by sodium dodecyl sulphate

SWV – square-wave voltammetry

WO_3_/SPE - screen printed electrode modified by WO_3_ rods


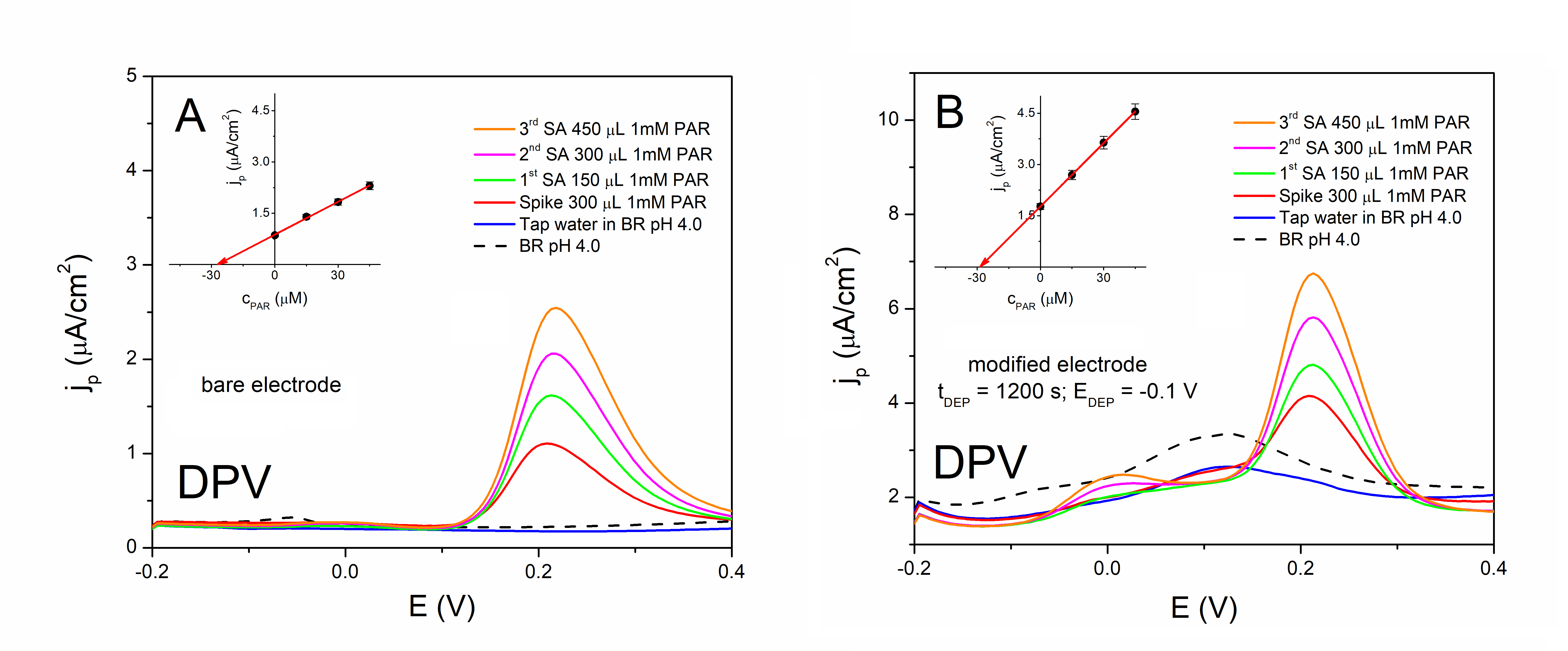


**Fig. S20 –** DP voltammograms of the analysis of „spiked“ tap water, consecutives standard additions of 1 mM PAR (1^st^ SA, 2^nd^ SA, 3^rd^ SA) with corresponding graphical evaluation of PAR quantification by the standard addition method (inset) on bare SPCE **(A)** and manually prepared AuNPs-SPCE **(B)**. Pulse parameters: pulse height 100 mV, pulse time 100 ms and interval time 0.5 s.


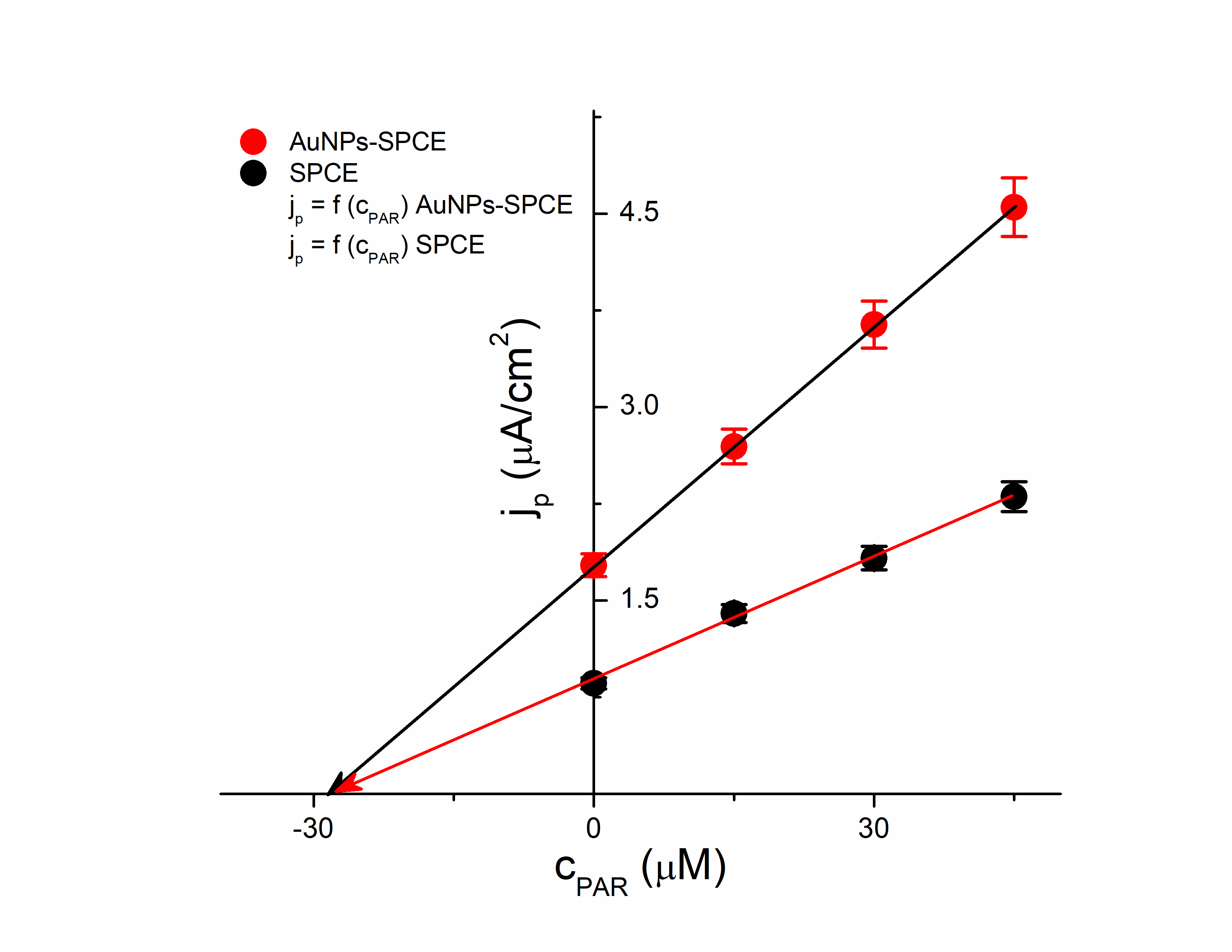


**Fig. S21 –** Comparison of graphical evaluation of PAR quantification by the standard addition method on bare SPCE and manually prepared AuNPs-SPCE.


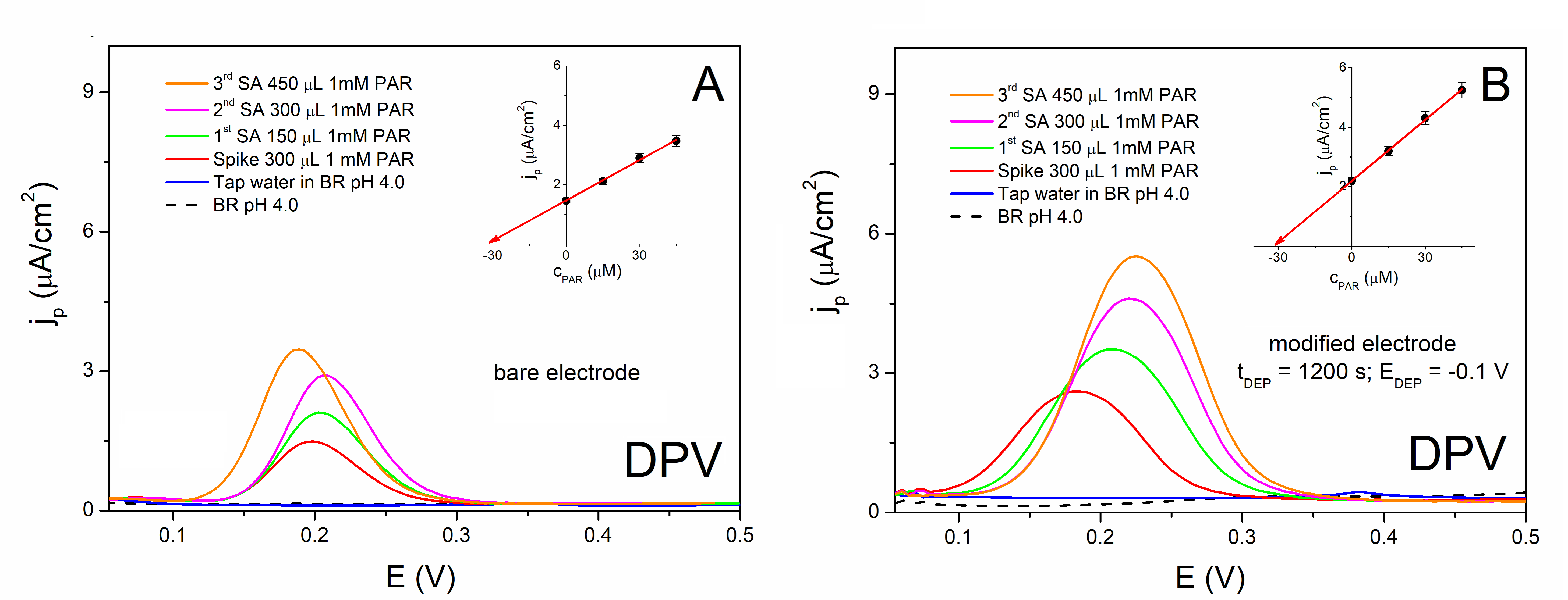


**Fig. S22 –** DP voltammograms of the analysis of „spiked“ tap water, consecutives standard additions of 1 mM PAR (1^st^ SA, 2^nd^ SA, 3^rd^ SA) with corresponding graphical evaluation of PAR quantification by the standard addition method (inset) on bare SPCE **(A)** and AuNPs-SPCE prepared by BES **(B)**. Pulse parameters: pulse height 100 mV, pulse time 100 ms and interval time 0.5 s.


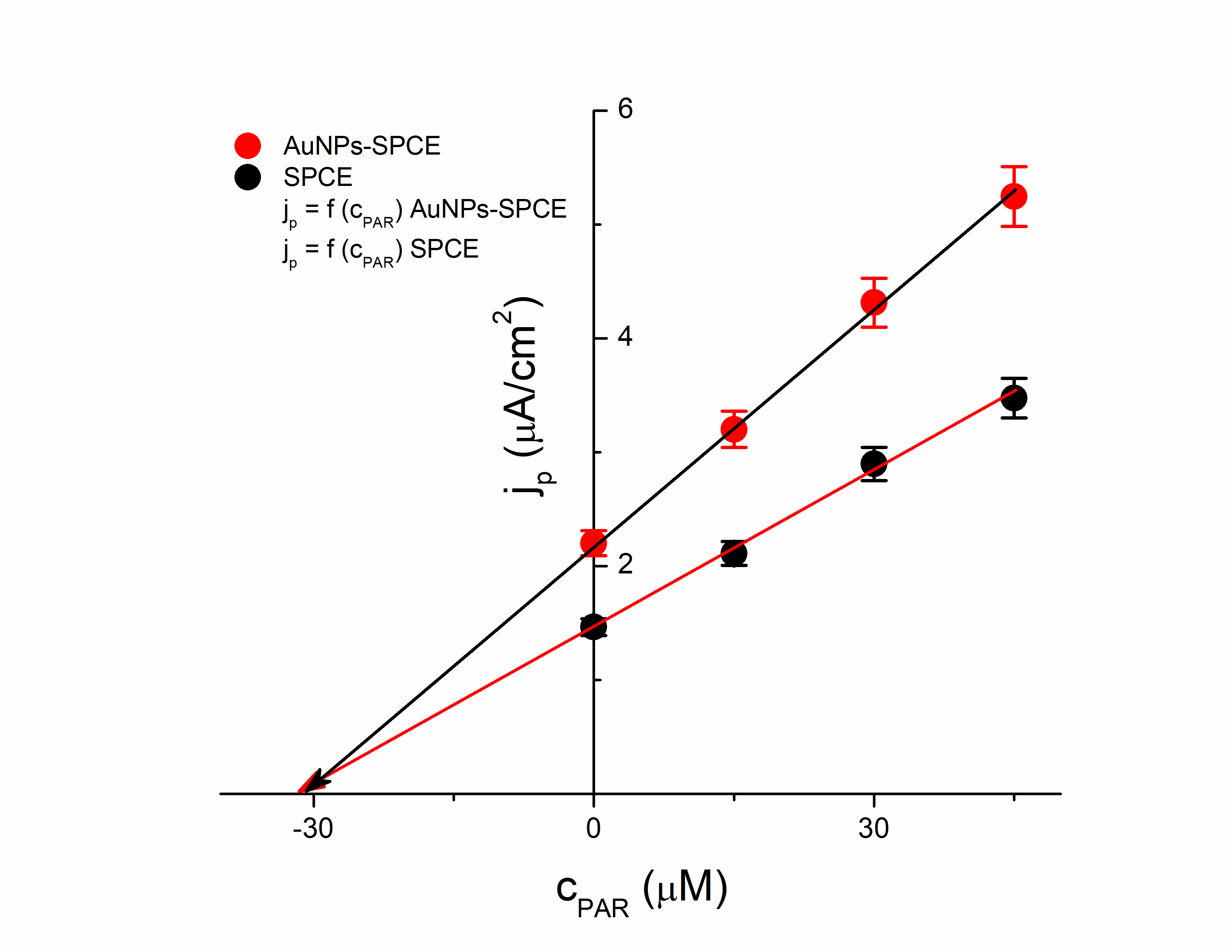


**Fig. S23 –** Comparison of graphical evaluation of PAR quantification by the standard addition method on bare SPCE and AuNPs-SPCE prepared by BES.


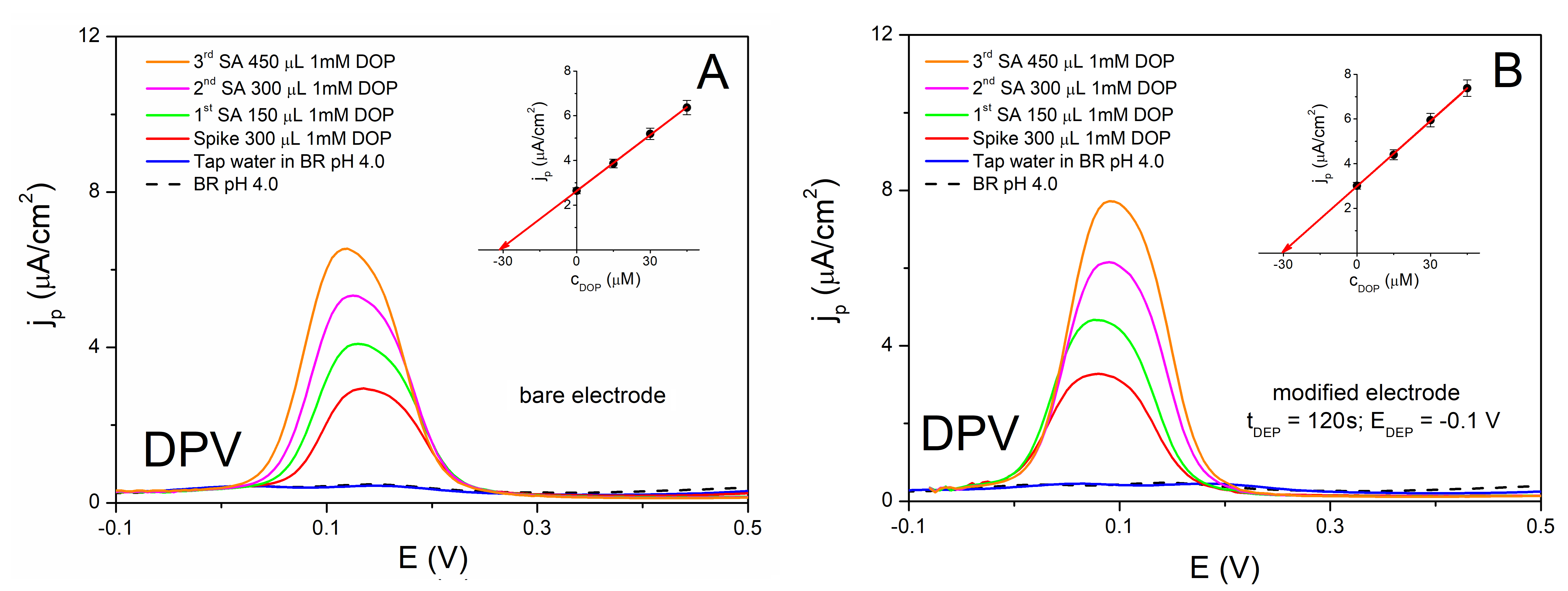


**Fig. S24 –** DP voltammograms of the analysis of „spiked“ tap water, consecutives standard additions of 1 mM DOP (1^st^ SA, 2^nd^ SA, 3^rd^ SA) with corresponding graphical evaluation of DOP quantification by the standard addition method (inset) on bare SPCE **(A)** and AuNPs-SPCE prepared by BES **(B)**. Pulse parameters: pulse height 100 mV, pulse time 100 ms and interval time 0.5 s.


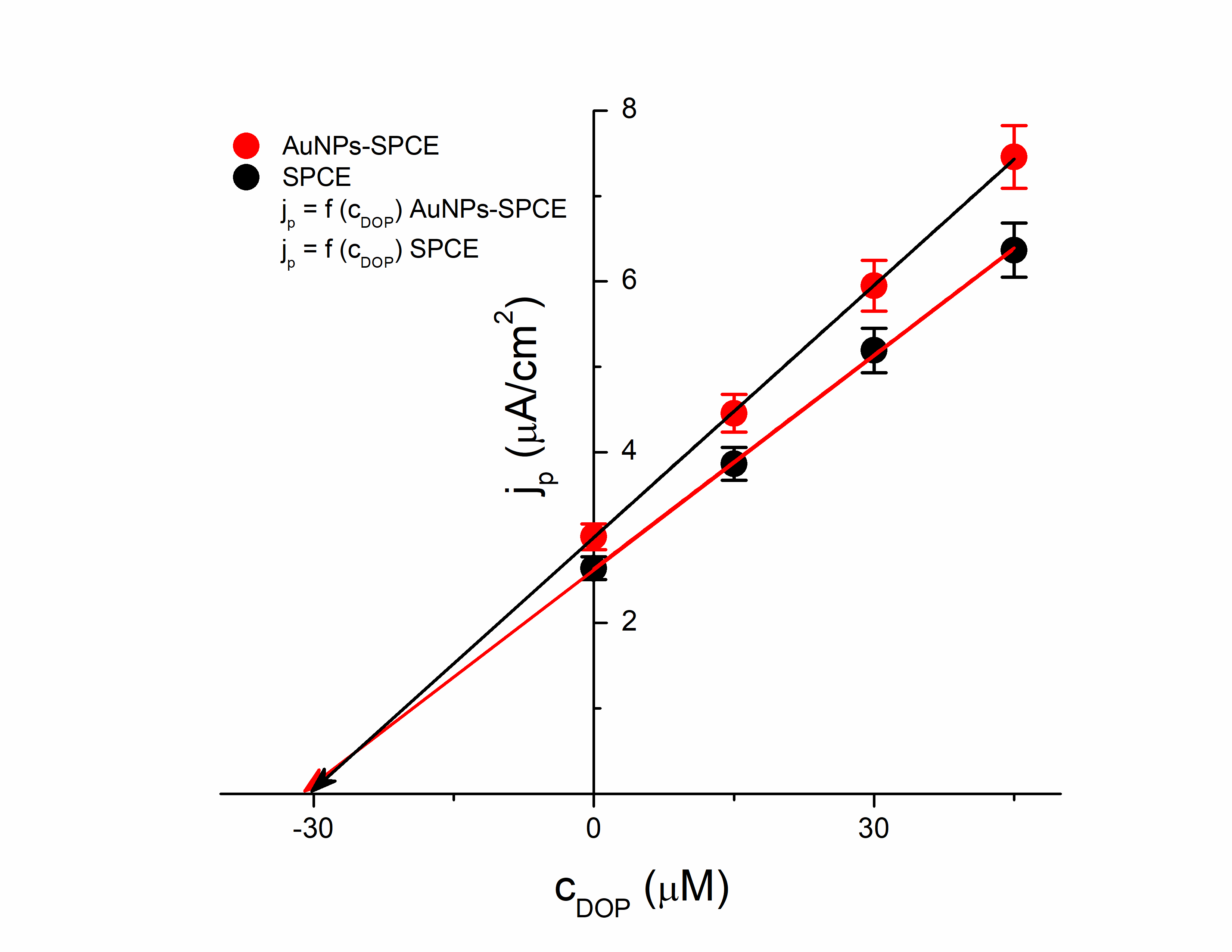


**Fig. S25 –** Comparison of graphical evaluation of DOP quantification by the standard addition method on bare SPCE and AuNPs-SPCE prepared by BES.

**Table T5**. – The determined amount of DOP in „spiked“ tap water by standard addition method for “spike-recovery” assay (*n* = 3).

| **Method of modification** | **Sensor** | **Sample/Matrix** | **DOP added (µM)** | **DOP measured (µM)** | **Recovery**  **(%)** |
| --- | --- | --- | --- | --- | --- |
| - | SPCE | Tap water | 30.0 | 31.0 ± 0.91 | 103.3 |
| BES | AuNPs-SPCE | Tap water | 30.0 | 30.3 ± 0.71 | 101.0 |


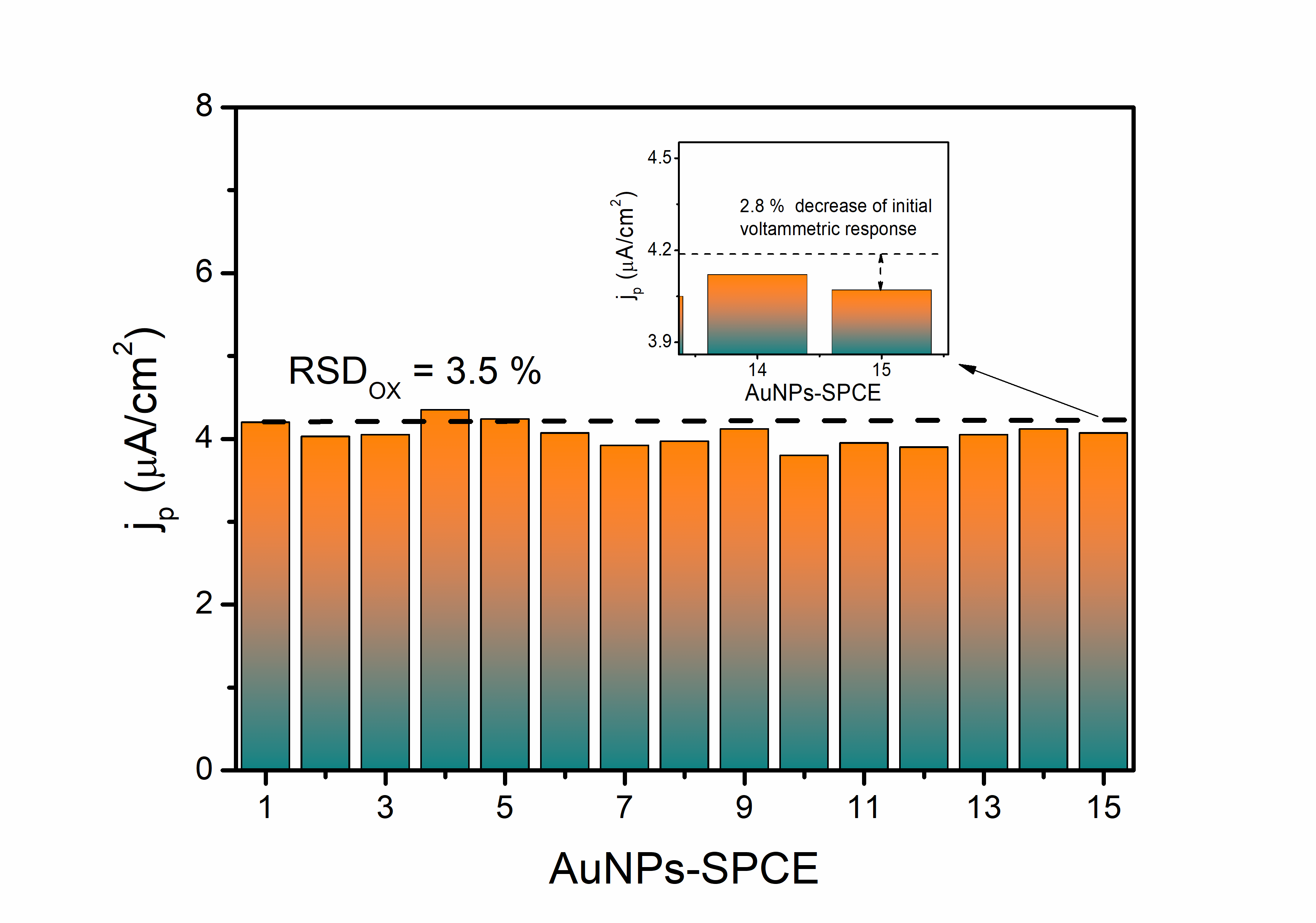


**Fig. S26 –** Recorded voltammetric responses of 50 µM DOP on fifteen AuNPs-SPCE at
*E*_DEP_ = –0.1 V and *t*_DEP_ = 300 s.
